# Buried Unstrained Germanium Channels: A Lattice‐Matched Platform for Quantum Technology

**DOI:** 10.1002/advs.202600066

**Published:** 2026-05-04

**Authors:** Davide Costa, Patrick Del Vecchio, Karina Hudson, Lucas E. A. Stehouwer, Alberto Tosato, Davide Degli Esposti, Vladimir Calvi, Luca Moreschini, Mario Lodari, Stefano Bosco, Giordano Scappucci

**Affiliations:** ^1^ QuTech and Kavli Institute of Nanoscience Delft University of Technology Lorentzweg Netherlands

**Keywords:** heterojunction, germanium, quantum technology, quantum transport

## Abstract

Strained germanium (ε‐Ge) and strained silicon (ε‐Si) buried quantum wells have enabled advanced spin‐qubit quantum processors. However, in the absence of suitable lattice‐matched substrates, ε‐Ge and ε‐Si are deposited on defective, metamorphic SiGe buffers, which may impact device performance and scaling. Here an alternative platform is introduced based on the heterojunction between bulk unstrained Ge and a lattice‐matched strained silicon‐germanium (ε‐SiGe) barrier, eliminating the need for metamorphic buffers altogether. In a structure with a 52‐nm‐thick ε‐SiGe barrier, a low‐disorder two‐dimensional hole gas is demonstrated with a high‐mobility of $1.33\times 10^{5}\;{\mathrm{cm}}^{2}/\mathrm{Vs}$ and a low percolation density of $1.4\left(1\right)\times 10^{10}\;{\mathrm{cm}}^{-2}$. Quantum transport shows that holes confined in the buried unstrained Ge channel have a strong density‐dependent in‐plane effective mass and out‐of‐plane g‐factor, pointing to a significant heavy‐hole–light‐hole mixing in agreement with theory. Measurements of Zeeman‐split levels in quantum point contacts further highlight this character, showing a two‐fold larger in‐plane g‐factor in Ge than in ε‐Ge. The prospects of strong spin–orbit interaction, isotopic purification, and of hosting superconducting pairing correlations make this platform appealing for fast quantum hardware and hybrid quantum systems.

## 1 Introduction

Continuous advances in materials underpin the development of semiconductor quantum technology [1] based on spin qubits in quantum dots [2] and superconductor–semiconductor hybrid devices [3]. Spin qubits were first realized in GaAs‐based heterostructures [4, 5], where lattice‐matched GaAs/AlGaAs epitaxy produced buried, high‐mobility electron gases and electrostatically defined quantum dots largely free of disorder [6]. However, the hyperfine interaction with the abundant nuclear spins in III–V materials strongly limited spin coherence [7], motivating a shift toward group‐IV semiconductors Si and Ge, which have a low natural abundance of nuclear spins and can be further isotopically purified [8, 9, 10]. In Si metal‐oxide‐semiconductor devices (Si‐MOS), isotopically purified Si epilayers are lattice matched to pristine, Si substrates [11, 12] and long spin coherence times have been demonstrated [13], while maintaining compatibility with advanced semiconductor manufacturing [14, 15]. Yet, qubits in Si‐MOS are defined at the semiconductor‐oxide interface, introducing electrostatic disorder and charge noise and posing a challenge for scaling [16].

Alternatively, spin‐qubits in strained Ge (ε‐Ge) [17, 18, 19] and strained Si (ε‐Si) [20, 21, 22, 23] buried quantum wells [24, 25] may experience a quiet electrical environment because the noisy semiconductor‐oxide interface is separated by an epitaxial SiGe barrier [26]. In the absence of high‐quality SiGe wafers for epitaxy, ε‐Ge and ε‐Si quantum wells are grown on strain‐relaxed SiGe buffers, which act as metamorphic substrates [27] bridging the lattice mismatch with the underlying Ge or Si wafers. However, these SiGe metamorphic substrates rely on networks of dislocations for strain‐release and are inherently defective, introducing topographic, strain, chemical, and band offset fluctuations in the strained quantum wells [28, 29, 30], thereby challenging the performance and cross‐wafer uniformity of quantum devices.

Here, we develop a group IV semiconductor heterostructure that has the potential to unite in a single material stack three key merits sought for spin qubits materials—buried channels for low electrostatic disorder, lattice matching to the substrate for a defect‐free crystal, and possibility of isotopic purification for long spin coherence–whereas preceding architectures offered only subsets of these advantages. The heterostructure is based on the heterojunction between unstrained Ge and a strained SiGe (ε‐SiGe) barrier that is lattice‐matched to a pristine Ge substrate, eliminating the need for metamorphic substrates. Building on the recent use of Ge wafers for SiGe heterostructures epitaxy [19, 31], this approach realizes a seminal but long‐overlooked design principle [32]: that two‐dimensional systems can be formed in elemental Ge by exploiting the band alignment of coherently strained SiGe barriers on Ge substrates.

These early oversimplified calculations [32] neglected the significant energy splitting between heavy‐holes (HH) and light holes (LH) due to quantum confinement [24, 33, 34] in the Ge channel at the heterojunction, leading to the challenging proposal of depositing highly strained ${\mathrm{Si}}_{0.5}$ ${\mathrm{Ge}}_{0.5}$ barriers to achieve sufficient band offset for confining a two‐dimensional hole gas (2DHG). This approach proved impractical in early experiments [35] and was soon abandoned in favor of ε‐Ge quantum wells on strain‐relaxed SiGe buffers [36]. Instead, from measurements on undoped insulated‐gate field‐effect transistors, supported by comprehensive self‐consistent Poisson–Schrödinger simulations, we demonstrate that even a moderately tensile‐strained ${\mathrm{Si}}_{0.2}$ ${\mathrm{Ge}}_{0.8}$ barrier provides sufficient band offset for robust confinement of a 2DHG at the buried heterojunction in Ge. The 2DHG has high mobility, low percolation density, and shows fractional quantum Hall states at low density. Quantum transport, supported by theoretical calculations, reveals electrically‐tunable in‐plane effective mass ($m^{*}$) and out‐of‐plane g‐factor ($g_{⊥}^{*}$), highlighting confinement dominated moderate HH–LH energy splitting leading to significant HH–LH mixing and enhanced spin–orbit interaction in unstrained Ge that marks a clear distinction from strain‐dominated HH‐LH large splittings in ε‐Ge quantum wells. This distinction is reinforced by further confinement into quantum point contacts, where the characterization of Zeeman‐split one dimensional subbands reveals a much larger in‐plane g‐factor ($g_{∥}^{*}$) in Ge compared to ε‐Ge.

## 2 Results and Discussion

**FIGURE 1:**
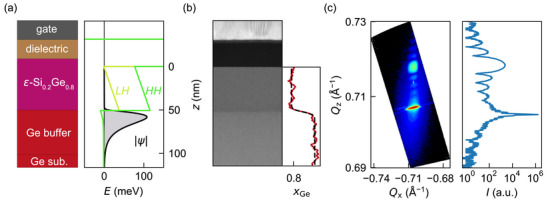
(a) Layer schematic of the semiconductor heterostructure and gate stack featuring an heterojunction between an unstrained Ge buffer and a strained SiGe (ε‐SiGe) barrier (left) and simulated band‐edges (right) with heavy holes (HH, green line), light holes (LH, light green line). The ground state heavy hole wavefunction |ψ| (black line) resides primarily in the Ge buffer, lattice‐matched to the Ge substrate. The Fermi energy is set as the reference energy at 0eV. (b) HAADF‐STEM image of the active layers of the Ge/ε‐SiGe heterostructure (left) with EDX profile (red line, right) showing the Ge alloy concentration ($x_{\mathrm{Ge}}$) as a red curve and fit to a sigmoid function (dotted black line). The vertical z‐axis scale is as in (a). (c) X‐ray diffraction reciprocal space map of the (–404) planes (left) as a function of the in‐plane ($Q_{x}$) and out‐of‐plane ($Q_{z}$) inverse of lattice spacing, with a high‐resolution ω/2θ scan around the Ge (004) peak (right). I is the signal intensity in arbitrary units.

The lattice‐matched Ge/ε‐SiGe heterostructure is grown by reduced‐pressure chemical vapour deposition on a 100mm Ge(001) wafer. As shown in Figure 1a (left panel), the semiconductor stack design comprises an unstrained 250nm epitaxial Ge buffer layer, a tensile‐strained 52nm ${\mathrm{Si}}_{0.2}$ ${\mathrm{Ge}}_{0.8}$ barrier, and a final sacrificial Si cap. Details of the epitaxy conditions for Ge and SiGe layers on Ge wafers are reported in [31]. Hall‐bar shaped heterostructure field effect transistors (H‐FETs) are fabricated with a low‐thermal budget process featuring platinum–germanosilicide ohmic contacts and an ${\mathrm{Al}}_{2}$ $O_{3}$/Ti/Pd gate stack as described in [37, 38]. Unlike defective metamorphic substrates, where strain relaxation is promoted by pre‐existing dislocations [39], growth on a pristine substrate allows for a sufficiently thick strained barrier to separate the heterojunction from the disordered dielectric, while still remaining below the theoretical critical thickness for strain relaxation [40, 41, 42].

One‐dimensional Schrödinger–Poisson simulations of the heavy‐hole (HH) and light‐hole (LH) band edges along the growth direction z are shown in the right panel of Figure 1a. The electric field from the insulated top‐gate induces a triangular quantum well at the buried Ge/ε‐${\mathrm{Si}}_{0.2}$ ${\mathrm{Ge}}_{0.8}$ heterojunction for accumulation of a 2DHG [43], advancing the theoretical understanding of these heterojunction presented in earlier work [32]. The HH wavefunction (|ψ|) resides predominantly in the unstrained Ge layer, where charge carrier confinement is promoted by a band‐offset of about 125meV at the heterojunction, arising from the strain‐induced splitting of the HH and LH bands in the ε‐${\mathrm{Si}}_{0.2}$ ${\mathrm{Ge}}_{0.8}$ layer and from quantum confinement of gate‐induced charge within the Ge layer. While the band offset is comparable to that in ε‐Ge quantum wells (∼130meV) [37], the HH–LH energy splitting is quite different. In this case, quantum confinement in the unstrained Ge layer yields a HH–LH splitting of about 3meV — much smaller than the 70meV typically observed in ε‐Ge. Nevertheless, this splitting remains sufficiently large to avoid the valley splitting challenge present for electrons in strained Si quantum wells [44, 45].

Figure 1b shows a high angle annular dark field (HAADF) scanning transmission electron microscopy (STEM) image of the active layers of the heterostructure, along with the energy dispersive X‐ray (EDX) profile of the Ge concentration $x_{\mathrm{Ge}}$. The image confirms the high‐quality epitaxial deposition of a 52(1)nm thick ${\mathrm{Si}}_{0.2}$ ${\mathrm{Ge}}_{0.8}$ barrier with no visible defects crossing the buried heterojunction. We estimate an upper bound for the characteristic length‐scale 4τ of the heterojunction interface of 3.8(3)nm by fitting the Ge content profile to a sigmoid model (see the Supporting Information).

As shown in the Supporting Information, characterization of the as‐grown heterostructure by atomic force microscopy and scanning Raman spectroscopy indicates that the ${\mathrm{Si}}_{0.2}$ ${\mathrm{Ge}}_{0.8}$ barrier is flat (root mean square roughness ∼0.4nm), tensile‐strained (average in‐plane strain $\bar{\varepsilon_{∥}}=1.0\left(4\right)\times 10^{-2}$), and exhibits no signs of a cross‐hatch pattern [46]. This marks a major difference compared to ε‐Ge (or ε‐Si) quantum wells, where the strain field associated with the underlying network of misfit dislocations in the strain‐relaxed buffer induces a prominent cross‐hatch pattern [29, 31, 45].

In Figure 1c (left panel), high resolution X‐ray diffraction reciprocal space mapping using the (–404) reflection shows that the ε‐${\mathrm{Si}}_{0.2}$ ${\mathrm{Ge}}_{0.8}$ and Ge peaks lie on the same vertical line. The position $Q_{x}$ of their lattice spacing in reciprocal space differs by only 0.07%, highlighting the similar in‐plane lattice constant and confirming the heterostructure is lattice‐matched. In the ω‐2θ scan around the Ge (004) peak (right panel), pronounced Pendellösung fringes indicate high crystalline quality with flat, parallel interfaces [47]. Analysis of their separation yields a 263.1(1)nm epitaxial Ge layer with a 52.7(5)nm ε‐${\mathrm{Si}}_{0.2}$ ${\mathrm{Ge}}_{0.8}$ barrier on top, in agreement with the intended design and HAADF‐STEM characterization.

**FIGURE 2:**
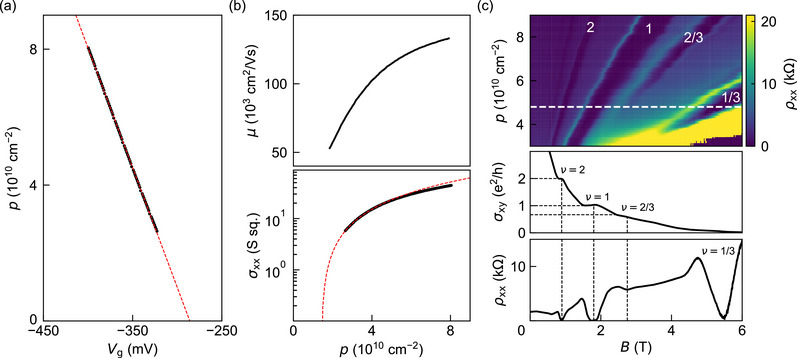
(a) Hall density (p) as a function of gate voltage ($V_{g}$) for a Ge/ε‐SiGe heterostructure field effect transistor (black curve) and corresponding linear fit (dashed red line). (b) Hole mobility (μ) in the top panel and longitudinal conductivity ($\sigma_{\mathrm{xx}}$) as black curve in the bottom panel as a function of p. The red dashed line is a fit to percolation theory in two‐dimensions. (c) Top panel: Landau fan diagram with longitudinal resistivity ($\rho_{\mathrm{xx}}$) as a function of perpendicular magnetic field B and p, obtained by stepping B and sweeping $V_{g}$. The dashed white line marks the density $p=4.8\times 10^{10}\;{\mathrm{cm}}^{-2}$ for detailed measurements of transversal conductivity $\sigma_{\mathrm{xy}}$ (central panel) and of $\rho_{\mathrm{xx}}$ (bottom panel). Quantum Hall plateaus and related Shubnikov–de Haas oscillation minima for integer and fractional states are highlighted with black dashed lines. All measurements are performed at a temperature of 60mK measured at the mixing chamber of the dilution refrigerator.

The electrical properties of the buried Ge/ε‐SiGe heterojunction are characterized by magnetotransport measurements of the H‐FET at a temperature of 60mK, using four‐terminal low‐frequency lock‐in techniques. Applying a negative gate voltage $V_{g}$ forms a 2DHG in accumulation mode with a tunable carrier density p. In the Supporting Information, we show the two‐terminal turn‐on curve of the H‐FET, measuring the source‐drain current as a function of $V_{g}$. The observed linear p‐$V_{g}$ relationship in Figure 2a (black curve) confirms a capacitively induced channel and excludes charge tunnelling into the SiGe LH states or toward the surface [48]. However, applying increasingly negative gate voltages above a density of $8.0\times 10^{10}\;{\mathrm{cm}}^{-2}$ causes a shift in the device characteristics due to charge trapping within the dielectric or at the semiconductor‐dielectric interface [49, 50], screening the further charge accumulation at the buried interface. From the fit (dashed red line) we estimate a capacitance per unit area C of $112.87\left(1\right)\;\mathrm{nF}/{\mathrm{cm}}^{2}$, in agreement with ε‐Ge quantum wells with similar barrier and dielectric thicknesses [38], indicating the 2DHG is formed at the buried heterojunction. Furthermore, we measure a minimum Hall density of $2.6\times 10^{10}\;{\mathrm{cm}}^{-2}$, on par with ε‐Ge quantum wells used for large spin qubit arrays [31, 38], hinting at a very low disorder channel.

The top and bottom panels in Figure 2b show the density‐dependent hole mobility μ(p) and longitudinal conductivity $\sigma_{\mathrm{xx}}\left(p\right)$, respectively. We measure a maximum mobility $\mu_{\max}$ of $1.33\times 10^{5}\;{\mathrm{cm}}^{2}/\mathrm{Vs}$ at a saturation density $p_{\mathrm{sat}}$ of $8.0\times 10^{10}\;{\mathrm{cm}}^{-2}$. Fitting the density‐dependent conductivity to 2D percolation theory, $\sigma_{\mathrm{xx}}\propto {\left(p-p_{p}\right)}^{1.31}$ [51, 52], we estimate a percolation‐induced critical density $p_{p}$ of $1.4\left(1\right)\times 10^{10}\;{\mathrm{cm}}^{-2}$, approaching the value of $1.22\left(3\right)\times 10^{10}\;{\mathrm{cm}}^{-2}$ achieved in ε‐Ge/SiGe quantum wells grown on Ge wafers with a similarly thick SiGe barrier [31]. This comparison suggests a similarly low‐disorder potential landscape at low densities, implying that quantum dots of about $1/\sqrt{p_{p}}∼80\;\mathrm{nm}$ in size, informative about the average distance between charge traps, are essentially disorder‐free [25]. However, the maximum mobility in Ge/ε‐SiGe is more than an order of magnitude lower than in ε‐Ge/SiGe. We speculate that the discrepancy in mobility at high density arises from impurity scattering from unwanted oxygen accumulation at the Ge/ε‐SiGe interface [53, 54], as shown by the secondary ion mass spectrometry in the Supporting Information, and from interface roughness scattering [55] associated with the rather diffused Ge/ε‐SiGe interface. Starting from this proof‐of‐principle heterostructure, we expect to reduce oxygen incorporation in the Ge and SiGe films by installing chemical filters in the gas precursor lines, leading to a potential mobility improvement up to 4× [54], or by refining the growth temperature profile during epitaxy [56, 57]. Furthermore, as discussed below, the heavier mass associated with HH–LH mixing at the higher end of the investigated density range contributes significantly to the observed mobility difference with ε‐Ge/SiGe quantum wells. A supplementary comparison of mobility, percolation density, and transport scattering time across group‐IV platforms for spin qubits is provided in the Supporting Information. This comparison shows that Ge/ε‐SiGe already significantly outperforms ε‐Si/SiGe and Si‐MOS when benchmarked in the low carrier density regime ($<1\times 10^{11}\;{\mathrm{cm}}^{-2}$) relevant for quantum dot qubit operation.

We further highlight the low‐disorder properties of the 2DHG by performing quantum transport measurements at higher perpendicular magnetic fields. The Landau level fan diagram in the top panel of Figure 2c shows $\rho_{\mathrm{xx}}$ as a function of perpendicular B and p. This has been calculated from the measurement of $\rho_{\mathrm{xx}}$ of a function of sweeping $V_{g}$ and stepping perpendicular B as shown in the Supporting Information. Dark blue regions correspond to dips in $\rho_{\mathrm{xx}}$ and highlight the density‐dependent evolution of integer and fractional filling factors ν=1/3,2/3,1,2, which fan out toward higher magnetic field and density. The dashed white line in the fan diagram indicates the magnetic field range selected for higher resolution measurements of $\rho_{\mathrm{xx}}$ and the transversal conductivity $\sigma_{\mathrm{xy}}$ at a fixed density of $p=4.8\times 10^{10}\;{\mathrm{cm}}^{-2}$, as shown in bottom and central panels of Figure 2c, respectively. A highlight of these measurements is the clear dip in $\rho_{\mathrm{xx}}$ corresponding to ν=1/3, a fractional quantum Hall state previously observed in lightly‐strained Ge quantum wells with hole mobility exceeding one million ${\mathrm{cm}}^{2}$/Vs [49] and relevant to the direct observation of anionic braiding statistics in GaAs [58].

**FIGURE 3:**
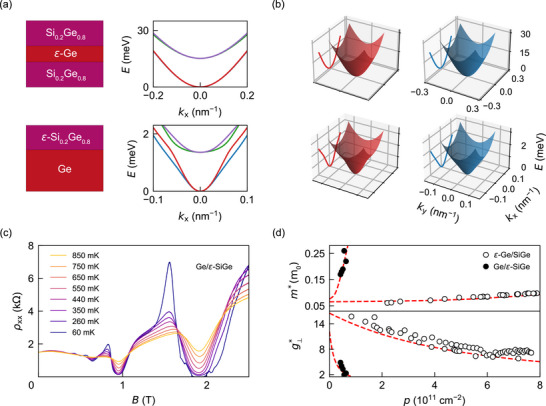
(a) Simulation of the first four energy levels at zero magnetic field and $k_{y}=0$ for the 2DHG in the strained Ge (ε‐Ge) quantum well (top, four HH levels) and in the unstrained Ge at the Ge/ε‐SiGe strained barrier heterojunction (bottom, two HH levels, blue and red, and two LH levels, green and purple). (b) Corresponding simulated dispersion relation of the spin up (red) and spin down (blue) ground state of the ε‐Ge quantum well (top) and of the unstrained Ge channel in the Ge/ε‐SiGe heterojunction (bottom). (c) Longitudinal resistivity ($\rho_{\mathrm{xx}}$) as a function of perpendicular magnetic field B at Hall density $p=4.55\times 10^{10}\;{\mathrm{cm}}^{-2}$, measured at different temperatures, ranging from from 60mK (blue) to 850mK (yellow) and measured at the mixing chamber of the dilution refrigerator. (d) Extracted in‐plane effective mass ($m^{*}$) and effective out‐of‐plane g‐factor ($g_{⊥}^{*}$) values for the 2DHG in Ge/ε‐SiGe (filled circles) and in ε‐Ge/SiGe (open circles) with theoretical simulation of these parameters (dashed red lines).

We simulate the band structure of the Ge/ε‐SiGe strained‐barrier heterojunction and, as a reference, of the ε‐Ge quantum well including electric and magnetic fields (see Supporting Information) to evaluate and benchmark $m^{*}$ and $g_{⊥}^{*}$. These band structure parameters exhibit substantial variations between the two systems because of the large difference in HH–LH splitting. The simulated spin‐dependent energy dispersions at zero magnetic field are shown in Figure 3a,b.

As a reference, in ε‐Ge quantum wells, the HH–LH splitting is largely dominated by the compressive strain in Ge, which shifts the lowest LH level roughly 70meV above the HH ground state. This large separation leads to an HH energy dispersion that at low densities is mostly parabolic, spin‐independent, and with a small in‐plane effective mass [59, 60]. In contrast, in the unstrained Ge channel at the Ge/ε‐SiGe heterojunction, the HH‐LH energy splitting is ∼3meV and is caused by the electric field‐induced quantum confinement, which differs for HHs and LHs because of their different out‐of‐plane mass. In this case, the HH ground state dispersion shows a strong non‐parabolicity and spin‐dependence at densities comparable to the one measured in our H‐FETs ($k_{x}=0.1\;{\mathrm{nm}}^{-1}$ corresponds to $p∼10^{11}\;{\mathrm{cm}}^{-2}$), as seen in Figure 3a,b. The large and tunable HH–LH mixing in the ground state of the heterojunction leads to an increase of $m^{*}$ and a decrease of $g_{⊥}^{*}$ compared to the strained quantum well, in agreement with the measurements in our devices.

We estimate in‐plane $m^{*}$ and $g_{⊥}^{*}$ from the temperature‐dependent decay of the Shubnikov–de Haas oscillation resistivity $\rho_{\mathrm{xx}}$ minima for different integer filling factors $\nu =ph/eB_{\nu }$, where $B_{\nu }$ is the magnetic field at integer ν. Figure 3c shows, for the H‐FET discussed in Figure 2, an exemplary dataset comprising magnetoresistivity $\rho_{\mathrm{xx}}\left(B\right)$ curves measured at a fixed density ($p=4.55\times 10^{10}\;{\mathrm{cm}}^{-2}$) for different temperature T in the 60 to 850mK range. Thermally activated Shubnikov–de Haas oscillations minima are visible at filling factors ν=1,2,3 from which we extract $m^{*}$ and $g_{⊥}^{*}$ according to the procedure in Ref. [49] and discussed in the Supporting Information. We repeat these measurements for five different densities from 4.2 to $6.3\times 10^{10}\;{\mathrm{cm}}^{-2}$ and plot the obtained density dependent $m^{*}$ and $g_{⊥}^{*}$ in Figure 3d (filled circles). At the lowest measured density ($4.2\times 10^{10}\;{\mathrm{cm}}^{-2}$) we obtain an effective mass of $0.17m_{0}$ and a $g_{⊥}^{*}$ of 4.85. We also report, as a comparison, previous data from ε‐Ge quantum wells [59, 61] (open circles).

In both systems, the measured trends are in satisfactory agreement with our theoretical predictions based on Landau levels simulations (Supporting Information). At a fixed density, holes confined in the Ge/ε‐SiGe heterojunction have a larger $m^{*}$ and smaller $g_{⊥}^{*}$ compared to the ε‐Ge quantum well, with a more pronounced sensitivity to the change in density caused by electric fields. This behavior arises from the reduced HH–LH energy splitting in the Ge/ε‐SiGe heterojunction, which leads to an enhanced and density‐dependent HH–LH mixing that increases at larger densities.

**FIGURE 4:**
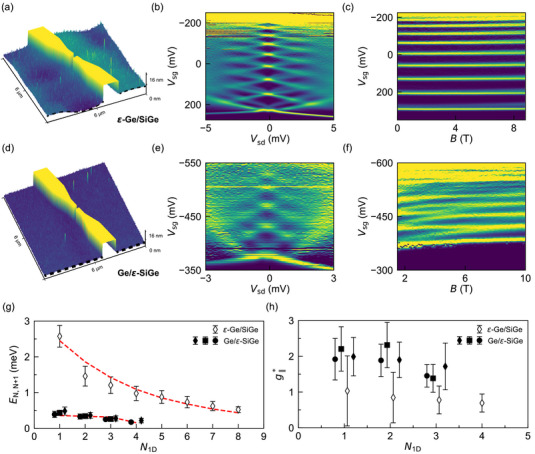
(a), (d) Atomic force microscopy (AFM) images of quantum point contact (QPC) devices fabricated on ε‐Ge/SiGe and on Ge/ε‐SiGe heterostructures, showing the device constriction side gates and the absence of cross‐hatch pattern in the lattice‐matched platform. (b),(e) Source–drain bias spectroscopy of the differential transconductance ($\partial G_{\mathrm{xx}}/\partial V_{\mathrm{sg}}$) as a function of side‐gate voltage ($V_{\mathrm{sg}}$) and source–drain bias ($V_{\mathrm{sd}}$) at a density of $8\times 10^{10}\;{\mathrm{cm}}^{-2}$, revealing clear 1D subband quantization. The asymmetry with respect to zero bias is due to a DC voltage offset of ∼0.1 mV in our measurement electronics. (c),(f) Zeeman spectroscopy of the QPCs at a density of $8\times 10^{10}\;{\mathrm{cm}}^{-2}$, showing the evolution of spin‐resolved 1D subbands with in‐plane magnetic field B. (g) Extracted 1D subband spacings ($E_{N,N+1}$) from (b),(e) as a function of subband index (N) for the ε‐Ge quantum well (white points, measured at Hall density of $8\times 10^{10}\;{\mathrm{cm}}^{-2}$) and the unstrained Ge channel at the Ge/ε‐SiGe heterojunction (black points, measured at Hall density of $\left(4,6,8\right)\times 10^{10}\;{\mathrm{cm}}^{-2}$). (h) Effective in‐plane g‐factor ($g_{∥}^{*}$) as a function of N for both platforms at the same densities, extracted from the Zeeman splitting in (c), (f).

To extend the investigation of the electronic and spin properties of these HH–LH mixed states, we fabricated quantum point contacts (QPCs) using the same low‐thermal‐budget process employed for the H‐FETs. The further quantum confinement offered by these devices serves as a proxy for the future realization of quantum dots on this novel platform. Figure 4 ad shows representative atomic force microscopy (AFM) images of QPC devices realized on ε‐Ge/SiGe quantum wells and on the same Ge/ε‐SiGe heterojunction characterized for quantum transport. An insulated global top accumulation gate (not shown) induces a 2DHG of density $p_{2D}$, which is subsequently laterally confined into a one‐dimensional channel by the two side gates. The lithographically defined 1D channels formed by the two side gates have lateral dimensions of ∼300nm×300nm, consistent with previous designs on Ge quantum wells [62], and ∼200nm×200nm, respectively. The lithographically smaller channel implemented on the Ge/ε‐SiGe heterojunction provides stronger lateral confinement, beneficial to effectively confine the expected heavier carriers. The AFM images highlight that the vertical undulation of the cross‐hatch pattern in ε‐Ge quantum wells has a length scale comparable to the size of the QPC nanoscale gate electrodes, potentially impacting device electrostatics. Instead, this undulation is absent in the lattice‐matched Ge/ε‐SiGe platform, providing a smooth and featureless template for nanofabrication.

We observe quantized conductance plateaus as a function of side‐gate voltage, indicative of ballistic transport in both material platforms, as shown in the Supporting Information. Source–drain bias spectroscopy of the differential transconductance $\partial G_{\mathrm{xx}}/\partial V_{\mathrm{sg}}$ as a function of the side‐gate voltage $V_{\mathrm{sg}}$ and source–drain bias $V_{\mathrm{sd}}$ (Figure 4b–e) reveals clear 1D subband quantization in both ε‐Ge/SiGe and Ge/ε‐SiGe. The corresponding 1D subband energy spacings $E_{N,N+1}$ are extracted from these measurements by evaluating the gate lever arm from the slopes of the transconductance diamond edges following the procedure described in Ref. [62] and are shown in Figure 4g as a function of subband index N. Also displayed are simulations of the subband energy spacings from adjusted in‐plane confinement profiles and identical heterostructure parameters as in the calculation of the 2DHG confined within the heterojunction plane. For the unstrained Ge channel at the Ge/ε‐SiGe heterojunction, we measured three different Hall densities of $\left(4,6,8\right)\times 10^{10}\;{\mathrm{cm}}^{-2}$ and the subband energy spacings are in good agreement with those computed from a simple parabolic model with characteristic length ℓ=28nm for all analyzed densities. For the ε‐Ge quantum well, measured at a density $p_{2D}=8\times 10^{10}\;{\mathrm{cm}}^{-2}$, the spacings become smaller with N, indicating a weaker confinement strength for excited subbands. In this case, the experimental spacings are in good agreement with a confinement profile of effective length ℓ=9.9nm and barrier height $V_{0}=13.5\;\mathrm{meV}$ (see Supporting Information). These values are also consistent with those reported for ε‐Ge quantum wells on Si wafers [62], which confirms the quality and reproducibility of Ge quantum point contacts. The reduced values observed in the unstrained heterojunction reflect the expected heavier effective mass.

Zeeman spectroscopy of the QPCs Figure 4c–f), shows the corresponding evolution of spin‐resolved 1D subbands with in‐plane magnetic field B. From these measurements we evaluate the effective in‐plane g‐factor $g_{∥}^{*}$, at the same densities considered in the subband energy spectroscopy. The in‐plane g‐factor is a key parameter for electrically driven spin‐qubit operation in current hole‐based quantum computing schemes, because it sets the Zeeman splitting and thus the qubit resonance condition for electric‐dipole spin‐resonance (EDSR) driving. As summarized in Figure 4h, the unstrained Ge QPC in Ge/ε‐SiGe exhibits higher $g_{∥}^{*}$ values compared to ε‐Ge/SiGe, consistent with the enhanced heavy‐hole–light‐hole mixing discussed above. The substantial error bars for the strained Ge quantum well g‐factor arise because its near‐zero in‐plane g‐factor produces minimal subband splitting, making the extraction uncertainty highly sensitive to intrinsic band broadening. For this first estimate, $g_{∥}^{*}$ values are extracted assuming a zero Zeeman splitting at B=0 T, growing linearly as a function of in‐plane magnetic field. A quantitative agreement between experiment and theory is presented in the Supporting Information, where we account for the complex magnetic field dependence of $g_{∥}^{*}$, arising from the richer valence band structure of Ge compared to ε‐Ge quantum wells.

## 3 Conclusions

In conclusion, we have introduced and experimentally validated a group IV semiconductor platform that hosts a high‐quality buried channel in a defect‐free crystalline host environment. Being lattice‐matched to the Ge substrate, our approach eliminates the need for strained relaxed buffer layers, which is promising for improving the homogeneity of future quantum dot devices built on this platform toward scalable quantum computing architectures. The absence of sizeable fluctuations of strain, and consequently band‐offset, in the Ge/εSiGe heterostructure results in a heightened susceptibility of bandstructure parameters to external electric fields, offering avenues for quantum engineering in a low‐disorder, dislocation‐free planar platform. Further tuning of the deposition parameters is expected to improve the disorder properties of the 2DHG, which already sets a benchmark for lattice‐matched material stacks in group IV semiconductor, such as electrons in Si‐MOS [63, 64]. The strong HH–LH mixing, induced by the rich valence band structure, induces in 2DHGs a tunable out‐of‐plane g‐factor and in‐plane effective mass, which stays light in the limit of small densities. Further confining to QPCs highlights the strong admixture of HH and LH, with smaller subband energies and larger $g_{∥}^{*}$ in Ge than in ε‐Ge, consistent with theoretical expectations.

Unstrained Ge layers hold promise for hole spin qubits, with significantly enhanced Rabi frequencies and quality factors predicted in comparison to ε‐Ge quantum wells [65, 66, 67]. The enhanced spin–orbit coupling expected in this low‐disorder system, along with the potential to host superconducting pairing correlations and the observation of fractional quantum Hall states, make this dislocation‐free Ge platform promising for fast quantum hardware based on spin qubits, hybrid quantum systems based on semiconductor‐superconductor quantum devices and fundamental condensed matter physics studies.

## Conflicts of Interest

G.S., A.T., and L.E.A.S. are inventors on a patent application (International Application No. PCT/NL2024/050178) submitted by Delft University of Technology related to devices in the lattice‐matched Ge/SiGe heterojunction. G.S. is founding advisor of Groove Quantum BV and declares equity interests.

## Supporting information


**Supporting File**: advs75421‐sup‐0001‐SuppMat.pdf.

## Data Availability

The data that support the findings of this study are openly available in [Data Repository for Buried unstrained Ge channels: a lattice‐matched platform for quantum technology] at [https://doi.org/10.5281/zenodo.15592817], reference number [61].

## References

[1] N. P. De Leon , K. M. Itoh , D. Kim et al., “Materials Challenges and Opportunities for Quantum Computing Hardware”, Science 372 (2021): abb2823. 10.1126/science.abb2823 33859004

[2] G. Burkard , T. D. Ladd , A. Pan , J. M. Nichol , and J. R. Petta , “Semiconductor Spin Qubits”, Review of Modern Physics 95 (2023): 025003.

[3] E. Prada , P. San‐Jose , M. W. A. De Moor , et al., “From Andreev to Majorana Bound States in Hybrid Superconductor–semiconductor Nanowires”, Nature Reviews Physics 2 (2020): 575.

[4] J. R. Petta , A. C. Johnson , J. M. Taylor , et al., “Coherent Manipulation of Coupled Electron Spins in Semiconductor Quantum Dots,” Science 309 (2005): 2180. 16141370 10.1126/science.1116955

[5] F. H. L. Koppens , C. Buizert , K. J. Tielrooij , et al., “Driven Coherent Oscillations of a Single Electron Spin in a Quantum Dot”, Nature 442 (2006): 766. 16915280 10.1038/nature05065

[6] R. Hanson , L. P. Kouwenhoven , J. R. Petta , S. Tarucha , and L. M. K. Vandersypen , “Spins in Few‐electron Quantum Dots”, Reviews of Modern Physics 79 (2007): 1217.

[7] L. Cywiński , W. M. Witzel , and S. Das Sarma , “Electron Spin Dephasing due to Hyperfine Interactions with a Nuclear Spin Bath”, Physical Review Letters 102 (2009): 057601. 19257553 10.1103/PhysRevLett.102.057601

[8] K. M. Itoh , W. L. Hansen , E. E. Haller , et al., “High Purity Isotopically Enriched 70Ge and 74Ge Single Crystals: Isotope Separation, Growth, and Properties”, Journal of Materials Research 8 (1993): 1341.

[9] A. Saraiva , W. H. Lim , C. H. Yang , C. C. Escott , A. Laucht , and A. S. Dzurak , “Materials for Silicon Quantum Dots and Their Impact on Electron Spin Qubits”, Advanced Functional Materials 32 (2022): 2105488.

[10] O. Moutanabbir , S. Assali , A. Attiaoui , et al., “Nuclear Spin‐Depleted, Isotopically Enriched 70Ge/28Si70Ge Quantum Wells”, Advanced Materials 36 (2024): 2305703. 10.1002/adma.202305703 38009242

[11] S. Fukatsu , T. Takahashi , K. M. Itoh , et al., “Effect of the Si/SiO2 Interface on Self‐diffusion of Si in Semiconductor‐grade SiO2”, Applied Physics Letters 83 (2003): 3897.

[12] D. Sabbagh , N. Thomas , J. Torres , et al., “Quantum Transport Properties of Industrial 28Si/28SiO2”, Physical Review Applied 12 (2019): 014013.

[13] M. Veldhorst , J. C. C. Hwang , C. H. Yang , et al., “An Addressable Quantum Dot Qubit with Fault‐tolerant Control‐fidelity”, Nature Nanotechnology 9 (2014): 981. 10.1038/nnano.2014.216 25305743

[14] A. Zwerver , T. Krähenmann , T. Watson , et al., “Qubits Made by Advanced Semiconductor Manufacturing”, Nature Electronics 5 (2022): 184.

[15] P. Steinacker , N. Dumoulin Stuyck , W. H. Lim , et al., “Industry‐compatible Silicon Spin‐qubit Unit Cells Exceeding 99% Fidelity”, Nature 646 (2025): 81. 40993388 10.1038/s41586-025-09531-9 PMC12488496

[16] J. D. Cifuentes , T. Tanttu , W. Gilbert , et al., “Bounds to Electron Spin Qubit Variability for Scalable CMOS Architectures”, Nature Communications 15 (2024): 4299. 10.1038/s41467-024-48557-x PMC11106088 38769086

[17] N. W. Hendrickx , W. I. L. Lawrie , M. Russ , et al., “A Four‐qubit Germanium Quantum Processor”, Nature 591 (2021): 580. 33762771 10.1038/s41586-021-03332-6

[18] N. W. Hendrickx , L. Massai , M. Mergenthaler , et al., “Sweet‐spot Operation of a Germanium Hole Spin Qubit with Highly Anisotropic Noise Sensitivity”, Nature Materials 23 (2024): 920. 38760518 10.1038/s41563-024-01857-5 PMC11230914

[19] L. E. A. Stehouwer , C. X. Yu , B. van Straaten , et al., “Exploiting Strained Epitaxial Germanium for Scaling Low‐noise Spin Qubits at the Micrometre Scale”, Nature Materials (2025): 1. 40619566 10.1038/s41563-025-02276-w PMC12657214

[20] J. Yoneda , K. Takeda , T. Otsuka , et al., “A Quantum‐dot Spin Qubit with Coherence Limited by Charge Noise and Fidelity Higher than 99.9%”, Nature Nanotechnology 13 (2018): 102. 10.1038/s41565-017-0014-x 29255292

[21] X. Xue , M. Russ , N. Samkharadze , et al., “Quantum Logic with Spin Qubits Crossing the Surface Code Threshold”, Nature 601 (2022): 343. 35046604 10.1038/s41586-021-04273-w PMC8770146

[22] A. Noiri , K. Takeda , T. Nakajima , et al., “Fast Universal Quantum Gate above the Fault‐tolerance Threshold in Silicon”, Nature 601 (2022): 338. 35046603 10.1038/s41586-021-04182-y

[23] S. Neyens , O. K. Zietz , T. F. Watson , et al., “Probing Single Electrons across 300‐mm Spin Qubit Wafers”, Nature 629 (2024): 80. 38693414 10.1038/s41586-024-07275-6 PMC11062914

[24] G. Scappucci , C. Kloeffel , F. A. Zwanenburg , et al., “The germanium Quantum Information Route”, Nature Reviews Materials 6 (2021): 926.

[25] G. Scappucci , P. J. Taylor , J. R. Williams , T. Ginley , and S. Law , “Crystalline Materials for Quantum Computing: Semiconductor Heterostructures and Topological Insulators Exemplars”, MRS Bulletin 46 (2021): 596.

[26] B. Paquelet Wuetz , D. Degli Esposti , A.‐M. J. Zwerver , et al., “Reducing Charge Noise in Quantum Dots by Using Thin Silicon Quantum Wells”, Nature Communications 14 (2023): 1385. 10.1038/s41467-023-36951-w PMC10011559 36914637

[27] P. W. Deelman , L. F. Edge , and C. A. Jackson , “Metamorphic Materials for Quantum Computing”, MRS Bulletin 41 (2016): 224.

[28] P. G. Evans , D. E. Savage , J. R. Prance , et al., “Nanoscale Distortions of Si Quantum Wells in Si/SiGe Quantum‐Electronic Heterostructures”, Advanced Materials 24 (2012): 5217. 22806921 10.1002/adma.201201833

[29] C. Corley‐Wiciak , C. Richter , M. H. Zoellner , et al., “Nanoscale Mapping of the 3D Strain Tensor in a Germanium Quantum Well Hosting a Functional Spin Qubit Device”, ACS Applied Materials & Interfaces 15 (2023): 3119. 36598897 10.1021/acsami.2c17395 PMC9869329

[30] C. Corley‐Wiciak , M. Zoellner , I. Zaitsev , et al., “Lattice Deformation at Submicron Scale: X‐Ray Nanobeam Measurements of Elastic Strain in Electron Shuttling Devices”, Physical Review Applied 20 (2023): 024056.

[31] L. E. A. Stehouwer , A. Tosato , D. Degli Esposti , et al., “Germanium Wafers for Strained Quantum Wells with Low Disorder”, Applied Physics Letters 123 (2023): 092101.

[32] R. People , “Indirect Band Gap and Band Alignment for Coherently Strained Si(x)Ge(1−x) Bulk Alloys on Germanium (001) Substrates”, Physical Review B 34 (1986): 2508. 10.1103/physrevb.34.2508 9939944

[33] R. Winkler , M. Merkler , T. Darnhofer , and U. Rössler , “Theory for the Cyclotron Resonance of Holes in Strained Asymmetric Ge‐SiGe Quantum Wells”" Physical review B 53 (1996): 10858. 10.1103/physrevb.53.10858 9982656

[34] R. Winkler , Spin‐orbit Coupling Effects in Two‐Dimensional Electron and Hole Systems, Vol. 191 (Springer, 2003).

[35] G. R. Wagner and M. A. Janocko , “Observation of a Two‐dimensional Hole Gas in Boron‐doped Si0.5Ge0.5/Ge Heterostructures”, Applied Physics Letters 54 (1989): 66.

[36] E. Murakami , H. Etoh , K. Nakagawa , and M. Miyao , “High Hole Mobility in Modulation‐Doped and Strain‐Controlled p‐Si0.5Ge0.5/Ge/Si1‐XsGeXs Heterostructures Fabricated Using Molecular Beam Epitaxy”, Japanese Journal of Applied Physics 29 (1990): L1059.

[37] A. Sammak , D. Sabbagh , N. W. Hendrickx , et al., “Shallow and Undoped Germanium Quantum Wells: a Playground for Spin and Hybrid Quantum Technology”, Advanced Functional Materials 29 (2019): 1807613.

[38] M. Lodari , N. W. Hendrickx , W. I. L. Lawrie , et al., “Low Percolation Density and Charge Noise with Holes in Germanium”, Materials for Quantum Technology 1 (2021): 011002.

[39] J. Matthews and A. Blakeslee , “Defects in Epitaxial Multilayers: I. Misfit Dislocations”, Journal of Crystal Growth 27 (1974): 118.

[40] R. People and J. C. Bean , “Calculation of Critical Layer Thickness versus Lattice Mismatch for Ge(x)Si(1‐x)/Si Strained‐layer Heterostructures”, Applied Physics Letters 47 (1985): 322.

[41] J. C. Bean , “Strained‐Layer Epitaxy of Germanium‐Silicon Alloys”, Science 230 (1985): 127. 17842673 10.1126/science.230.4722.127

[42] M. M. Alam , Y. Wagatsuma , K. Okada , et al., “Critical Thickness of Strained Si(1‐x)Ge(x) on Ge(111) and Ge‐on‐Si(111)”, Applied Physics Express 12 (2019): 081005.

[43] G. Scappucci , A. Tosato , M. F. Russ , L. E. A. Stehouwer , and A. Sammak , “Method for Manufacturing a Single Heterojunction Semiconductor Device and such a Single Heterojunction Semiconductor Device”, Patent Cooperation Treaty (PCT) (2024).

[44] M. Friesen , M. A. Eriksson , and S. N. Coppersmith , “Magnetic Field Dependence of Valley Splitting in Realistic Si/SiGe Quantum Wells”, Applied Physics Letters 89 (2006): 202106.

[45] D. Degli Esposti , L. E. A. Stehouwer , O. Gül , et al., “Low Disorder and High Valley Splitting in Silicon”, npj Quantum Information 10 (2024), 10.1038/s41534-024-00826-9.

[46] M. H. Zoellner , M.‐I. Richard , G. A. Chahine , et al., “Imaging Structure and Composition Homogeneity of 300 Mm SiGe Virtual Substrates for Advanced CMOS Applications by Scanning X‐ray Diffraction Microscopy”, ACS Applied Materials & Interfaces 7 (2015): 9031. 25871429 10.1021/am508968b

[47] D. K. Bowen and B. K. Tanner , High resolution X‐ray diffractometry and topography (CRC press, 1998).

[48] Y.‐H. Su , Y. Chuang , C.‐Y. Liu , J.‐Y. Li , and T.‐M. Lu , “Effects of Surface Tunneling of Two‐dimensional Hole Gases in Undoped Ge/GeSi Heterostructures”, Physical Review Materials 1 (2017): 044601.

[49] M. Lodari , O. Kong , M. Rendell , et al., “Lightly Strained Germanium Quantum Wells with Hole Mobility Exceeding One Million”, Applied Physics Letters 120 (2022): 122104.

[50] L. Massai , B. Hetényi , M. Mergenthaler , et al., “Impact of Interface Traps on Charge Noise and Low‐density Transport Properties in Ge/SiGe Heterostructures”, Communications Materials 5 (2024): 151. 39157449 10.1038/s43246-024-00563-8 PMC11324522

[51] L. A. Tracy , E. H. Hwang , K. Eng , et al., “Observation of Percolation‐induced Two‐dimensional Metal‐insulator Transition in a Si MOSFET”, Physical Review B 79 (2009): 235307.

[52] R. Fogelholm , “The Conductivity of Large Percolation Network Samples”, Journal of Physics C: Solid State Physics 13 (1980): L571.

[53] X. Mi , T. M. Hazard , C. Payette , et al., “Magnetotransport Studies of Mobility Limiting Mechanisms in Undoped Si/SiGe Heterostructures”, Physical Review B 92 (2015): 035304.

[54] J. Lu , J. Li , H. Wang , et al., “Impact of Oxygen Concentration on Hole Mobility in Undoped Ge Quantum Wells”, Chinese Physics B (2026).

[55] D. Costa , L. E. A. Stehouwer , Y. Huang , et al., “Reducing Disorder in Ge Quantum Wells by Using Thick SiGe Barriers”, Applied Physics Letters 125 (2024): 222104.

[56] S. W. Bedell , S. Hart , S. Bangsaruntip , et al., “Low‐temperature Growth of Strained Germanium Quantum Wells for High Mobility Applications”, ECS Transactions 98 (2020): 215.

[57] M. Myronov , J. Kycia , P. Waldron , et al., “Holes Outperform Electrons in Group IV Semiconductor Materials”, Small Science 3 (2023): 2200094. 40212767 10.1002/smsc.202200094 PMC11936008

[58] J. Nakamura , S. Liang , G. C. Gardner , and M. J. Manfra , “Direct Observation of Anyonic Braiding Statistics”, Nature Physics 16 (2020): 931. 10.1038/s41467-022-27958-w PMC8763912 35039497

[59] M. Lodari , A. Tosato , D. Sabbagh , et al., “Light Effective Hole Mass in Undoped Ge/SiGe Quantum Wells”, Physical Review B 100 (2019): 041304.

[60] L. A. Terrazos , E. Marcellina , Z. Wang , et al., “Theory of Hole‐spin Qubits in Strained germanium Quantum Dots”, Physical Review B 103 (2021): 125201.

[61] D. Costa , “Data Repository for ‘Unstrained Germanium Channels: a Lattice‐matched Platform for Semiconductor Quantum Technology’”, Zenodo (2025). 10.1002/advs.202600066 PMC13335600 42080338

[62] K. Hudson , D. Costa , D. D. Esposti , L. E. Stehouwer , and G. Scappucci , “Conductance Plateaus at quantum Hall Integer Filling Factors in germanium Quantum Point Contacts”, Applied Physics Letters 128 (2026): 052103.

[63] T. N. Camenzind , A. Elsayed , F. A. Mohiyaddin , et al., “High Mobility SiMOSFETs Fabricated in a Full 300 Mm CMOS Process”, Materials for Quantum Technology 1 (2021): 041001.

[64] A. Elsayed , M. M. K. Shehata , C. Godfrin , et al., “Low Charge Noise Quantum Dots with Industrial CMOS Manufacturing”, npj Quantum Information 10 (2024): 1.

[65] S. Bosco , M. Benito , C. Adelsberger , and D. Loss , “Squeezed Hole Spin Qubits in Ge Quantum Dots with Ultrafast Gates at Low Power”, Physical Review B 104 (2021): 115425.

[66] A. Secchi , G. Forghieri , P. Bordone , D. Loss , S. Bosco , and F. Troiani , “Hole‐spin Qubits in germanium beyond the Single‐particle Regime”, arXiv preprint arXiv:2505.02449 (2025).

[67] L. Mauro , M. J. Rodriguez , E. A. Rodriguez‐Mena , and Y.‐M. Niquet , “Hole Spin Qubits in Unstrained Germanium Layers”, npj Quantum Information 11 (2025): 1.

