# Supplementary material for: Buried Unstrained Germanium Channels: A Lattice‐Matched Platform for Quantum Technology
Source: Adv Sci (Weinh). 2026 May 4;13(40):e00066. doi: 10.1002/advs.202600066 (PMC13335600; doi:10.1002/advs.202600066)
Supplement: Supplementary file 1 — Supporting File: advs75421‐sup‐0001‐SuppMat.pdf. [file ADVS-13-e00066-s001.pdf]

Supporting Information for "Buried unstrained germanium channels: a lattice-matched platform for quantum technology"

Davide Costa, Patrick Del Vecchio, Karina Hudson, Lucas E. A. Stehouwer, Alberto Tosato, Davide Degli Esposti, Vladimir Calvi, Luca Moreschini, Mario Lodari, Stefano Bosco, and Giordano Scappucci*

QuTech and Kavli Institute of Nanoscience, Delft University of Technology, Lorentzweg 1, 2628 CJ Delft, Netherlands

(Dated: April 17, 2026)

CONTENTS

Atomic force microscopy 2

Raman spectroscopy 3

Energy dispersive X-ray 4

Secondary ion mass spectroscopy 5

Experimental Methods 6
- Turn-on current 6
- Landau fan diagram 6
- 2D effective mass and out-of-plane g-factor 7
- Transport metrics comparison 8
- 1D quantization 10
- 1D in-plane g-factor 10

Theoretical Model 11
- Two-dimensional hole gas 11
- Landau levels 14
- 1D channels 15

References 18

ATOMIC FORCE MICROSCOPY

We perform a 2D atomic force microscopy (AFM) scan of the surface of the Ge/ε-SiGe strained-barrier heterojunction and of a reference ε-Ge/SiGe strained quantum well (QW) over a 16×16 μm^2 and a 20×20 μm^2 region, respectively. A 3D view of the two measurements is shown in Supplementary Fig. 1. The lattice-matched nature of the heterojunction is confirmed by the absence of cross-hatch pattern, which is in turn very visible on the surface of the heterostructure featuring the strained Ge QW. The extracted root mean square surface roughness values are ~ 0.4 nm and ~ 1.8 nm, respectively.

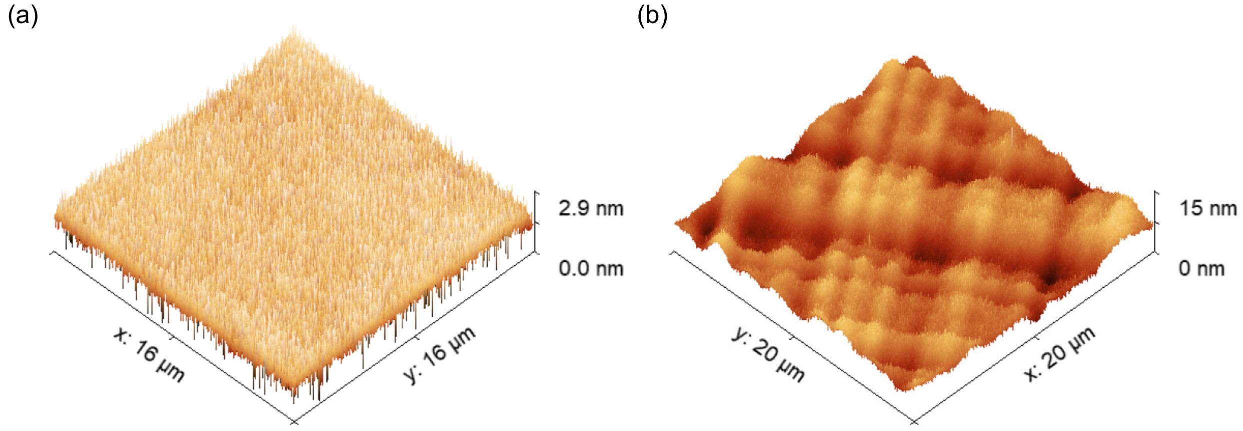

Supplementary Fig. 1. 3D AFM of (a) the Ge/ε-SiGe strained-barrier heterojunction and (b) the ε-Ge/SiGe strained quantum well.

RAMAN SPECTROSCOPY

We perform scanning Raman spectroscopy on both materials over a 20×20 µm^2 and a 15×15 µm^2 region, respectively. In particular, we extract the in-plane strain ε from the Ge-Ge vibration $\omega_{\mathrm{Ge-Ge}}$ in the Ge (Supplementary Fig. 2a) and SiGe (Supplementary Fig. 2b) layers of the Ge/ε-SiGe strained-barrier heterojunction and in the Ge layer of the ε-Ge/SiGe strained QW (Supplementary Fig. 2c). The mean strain values $\overline{\varepsilon}$ are -0.42×10^{-3} (no strain), 9.99×10^{-3} (tensile strain) and -7.45×10^{-3} (compressive strain), respectively. The larger strain in the ε-SiGe top barrier of the heterojunction arises from the lower Ge content (0.8) with respect to the SiGe barrier (0.83), setting the lattice parameter of the ε-Ge quantum wells. Moreover, the strain map of the ε-Ge quantum well shows signatures of the cross-hatch pattern, with regions featuring higher and lower strain around $\overline{\varepsilon}$, while the strain maps of the Ge/ε-SiGe strained-barrier heterojunction do not. We analyse the distribution of the normalized strain fluctuations $\Delta\varepsilon/\overline{\varepsilon}$, where $\Delta\varepsilon = \varepsilon - \overline{\varepsilon}$. The strain fluctuation distributions in the ε-SiGe layer of the Ge/ε-SiGe strained-barrier heterojunction (Supplementary Fig. 2e) and of the ε-Ge quantum well (Supplementary Fig. 2f) are quite similar and can be fitted to normal distributions (dashed black lines) whereas the fluctuation of the lattice-matched Ge layer in the Ge/ε-SiGe strained-barrier heterojunction (Supplementary Fig. 2d) have a much smaller probability density and can be therefore linked to measurement fluctuations.

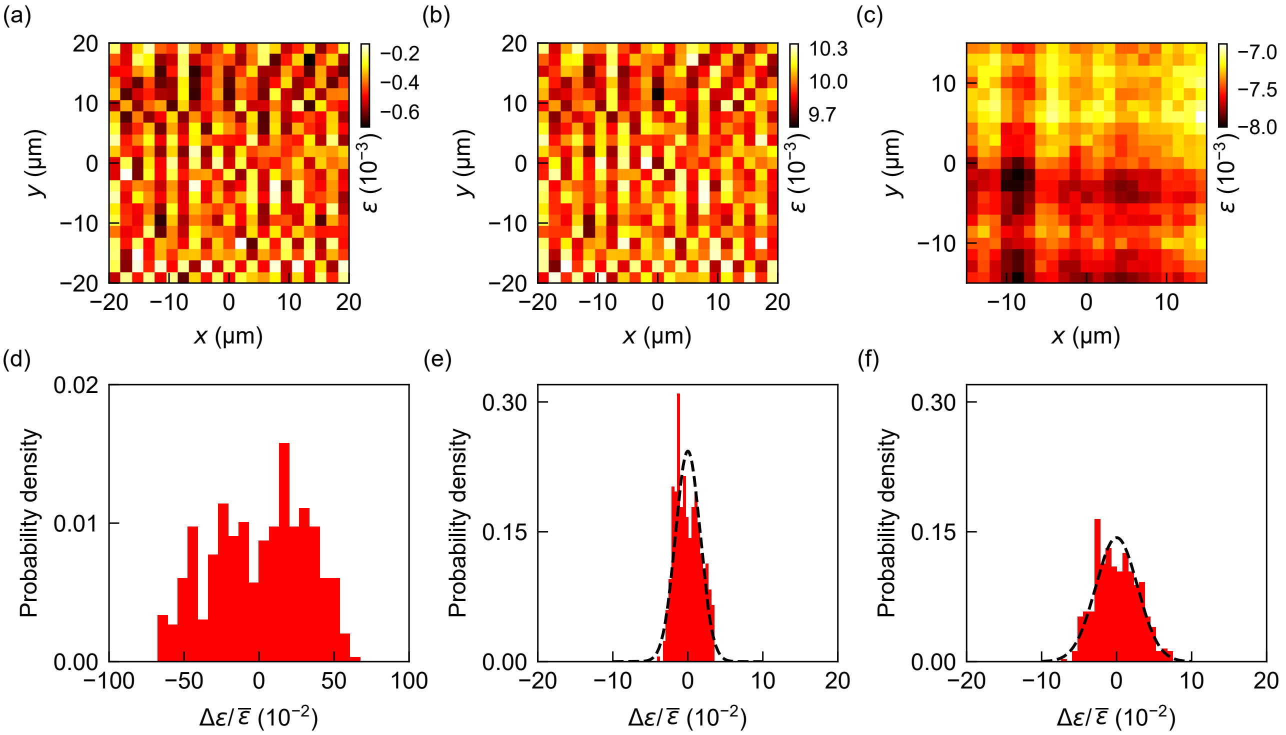

Supplementary Fig. 2. Raman strain maps corresponding to the ω_{Ge} Raman shifts in the Ge (a) and SiGe (b) layers of the Ge/ε-SiGe strained-barrier heterojunction and in the Ge layer of the ε-Ge QW (c). (d-e-f) Strain fluctuations from the Raman maps in (a-b-c), respectively, and normal distribution fit (dashed black line). Counts are normalized such that the area under the curve integrates to one.

ENERGY DISPERSIVE X-RAY

We perform an Energy Dispersive X-ray (EDX) scan of the Ge content x_{Ge} at the Ge/ε-SiGe strained-barrier heterojunction and fit it with the sigmoid function

$$\frac{1}{1+e^{\frac{x-x_0}{\tau}}}\,, \tag{1}$$

where x_0 is the position of the interface and τ is the characteristic length quantifying the heterojunction interface. The measurement and the sigmoid fit are shown in Supplementary Fig. 3, where $z = 0$ corresponds to the dielectric-semiconductor interface. We characterize the interface sharpness with the 4τ parameter corresponding to the length over which x_{Ge} changes from 0.12 to 0.88 of the asymptotic value, extracting a value of 3.8(3) nm. Due to convolution with the EDX interaction volume, this phenomenological value represents an upper bound rather than the true physical width.

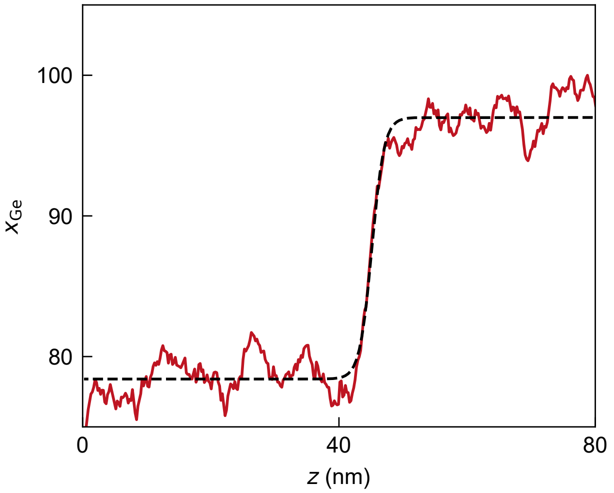

Supplementary Fig. 3. EDX scan of the Ge content at the Ge/ε-SiGe strained-barrier heterojunction with a sigmoid fit (dashed black line).

SECONDARY ION MASS SPECTROSCOPY

We analyse the chemical composition depth profile of the Ge/ε-SiGe strained-barrier heterojunction by secondary ion mass spectroscopy (SIMS) (Supplementary Fig. 4). The measurement shows an unwanted significant oxygen accumulation at the Ge/ε-SiGe (2×10^{18} at/cm^3) interface about 50 nm below the surface, which may be negatively impacting the channel performance.

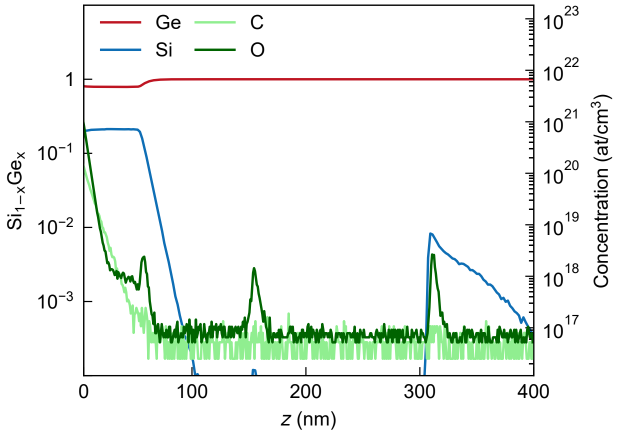

Supplementary Fig. 4. SIMS of the Ge/ε-SiGe strained-barrier heterojunction, showing Ge (red) and Si (blue) contents and O (dark green) and C (light green) concentrations.

EXPERIMENTAL METHODS

Turn-on current

The device exhibits a clear turn-on at approximately -290 mV, corresponding to the accumulation of the 2D hole gas in the unstrained Ge channel. The vertical dashed line indicates the start of the gate voltage range analyzed to extract mobilities and densities in the main text.

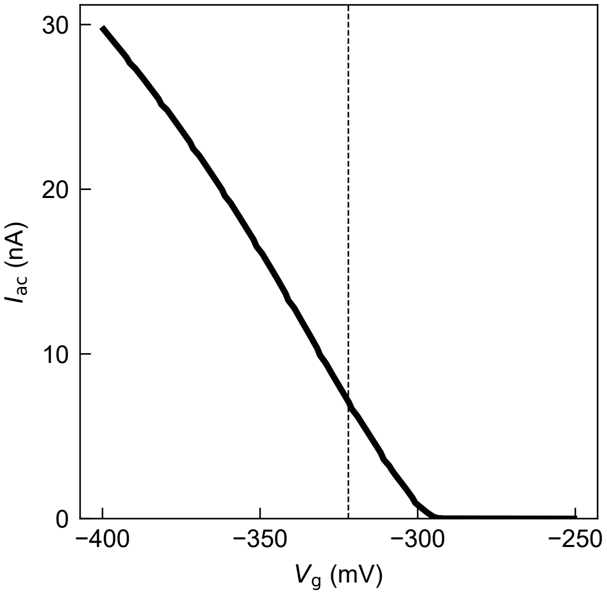

Supplementary Fig. 5. Source-drain AC current (I_{ac}) measured as a function of the applied gate voltage (V_g).

Landau fan diagram

By continuously sweeping the applied gate voltage V_g to tune the 2D hole gas density while stepping the perpendicular magnetic field B up to 6 T at a base temperature of 60 mK, we mapped the Landau fan diagram of the device. The resulting color map in Supplementary Fig. 6 clearly resolves the characteristic diagonal minima in ρ_{xx}, which correspond to the formation of quantized Landau levels discussed in the main text.

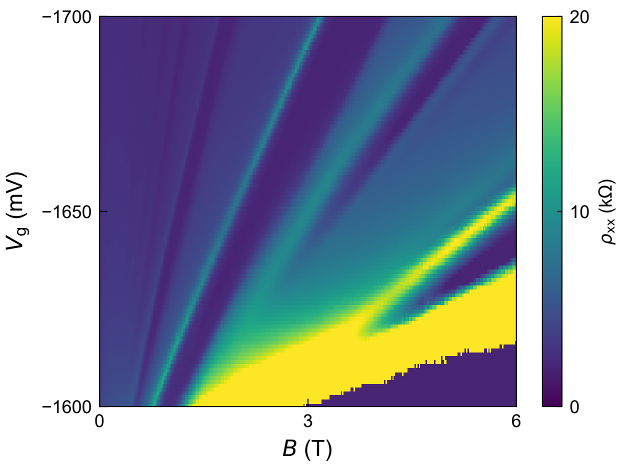

Supplementary Fig. 6. Color map of the longitudinal resistivity ρ_{xx} measured as a function of the perpendicular magnetic field B and the applied gate voltage V_{g}.

2D effective mass and out-of-plane g-factor

We extract the effective mass m^* and the out-of-plane g-factor $g^*_\perp$ from the main text exemplary dataset in Fig. 3c, using magnetoresistivity $\rho_{\mathrm{xx}}(B)$ curves measured at a fixed density ($p = 4.55 \times 10^{10}$ cm^{-2}) for different T (from 60 to 850 mK). The activation energy gap Δ_ν of each filling factor ν can be obtained from the thermally activated decay of the Shubnikov–de Haas oscillation resistivity ρ_{xx} minima for a given filling factor, as reported in the Arrhenius plot of the $\ln(\rho_{\mathrm{xx}})$ against T^{-1} (black circles, inset of Supplementary Fig. 7). Following the Boltzmann statistics, the longitudinal magnetoresistance of a specific minima can be described via the relation $\ln(\rho_{\mathrm{xx}}) \propto -\Delta_\nu/(2\mathrm{k_B}T)$, where $\mathrm{k_B}$ is the Boltzmann constant. Therefore, the activation energy of each filling factor can be extrapolated from the slope of a linear fit of the Arrhenius plot (dashed red line, inset of Supplementary Fig. 7). Since the even and odd filling factors correspond to the cyclotron frequency and the Zeeman splitting, respectively, and a linear relation links activation energy Δ_ν and the magnetic field B_ν at which each ν occurs, m^* and $g^*_\perp$ can be extrapolated from a linear fit of the $\Delta_\nu(B_\nu)$ dependence. Supplementary Fig. 7 shows the extrapolated activation energy Δ_ν as a function of magnetic field B for all the investigated even and odd filling factors (diamonds and circles, respectively). $g^*_\perp$ can be extrapolated from the slope of a linear fit $\Delta_{\nu,\mathrm{odd}} = g^*_\perp \mu_\mathrm{B} B - \Gamma$, where μ_B is the Bohr magneton and Γ is the disorder-induced Landau level broadening. Once g^* and Γ are estimated, m^* can be obtained from the slope of the linear fit $\Delta_{\nu,\mathrm{even}} = \hbar eB/m^* - g^*_\perp \mu_\mathrm{B} B - \Gamma$, where we fix $\Gamma = 134(4)$ μeV from the previous fit, due to the limited points. For the reference density p of 4.55×10^{10} cm^{-2}, we find $g^*_\perp = 4.0(1)$ and $m^* = 0.19(1)$.

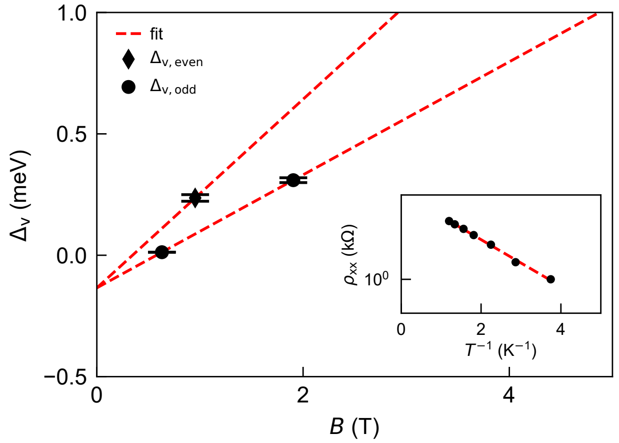

Supplementary Fig. 7. Activation energy gap Δ_v as a function of magnetic field B for even (diamonds) and odd (circles) filling factors ν, along with linear fits (dashed red lines). We assume the two lines have the same intercept at B=0, corresponding to the disorder-induced Landau level broadening Γ. The inset shows the Arrhenius plot and fit to extract Δ_v for $\nu = 5$.

Transport metrics comparison

Supplementary Fig. 8 benchmarks the electrical transport properties of the 2DHG in Ge/ε-SiGe (this study) against 2DHGs in ε-Ge/SiGe [1] and 2DEGs in ε-Si/SiGe [2] and Si-MOS [3]—state of the art material stacks that have supported functional spin-qubit devices.

The left panel shows the density dependent mobility. We benchmark at $p = 8 \times 10^{10}$ cm^{-2} (black dashed line), a convenient density at which three platforms can be directly compared and which remains relevant for quantum dot operation, corresponding to a realistic ∼40 nm diameter dot occupied by a single charge. At this density, holes in Ge/ε-SiGe achieve a mobility of $\sim 133 \times 10^3$ cm^2/Vs, nearly an order of magnitude higher than electrons in ε-Si/SiGe ($\sim 15 \times 10^3$ cm^2/Vs) [2]. Relative to Si-MOS [3], a lattice-matched platform free of cross-hatch defects, Ge/ε-SiGe again shows superior performance: transport in Si-MOS is not measurable at this density, and its peak mobility ($\sim 1.5 \times 10^4$ cm^2/Vs) remains an order of magnitude lower. Finally, the maximum mobility in Ge/ε-SiGe is much lower (30×) than that of highly optimized ε-Ge/SiGe quantum wells ($\sim 2.5 \times 10^6$ cm^2/Vs [1] at the same carrier density of 8×10^{10} cm^{-2}).

The density-dependent conductivity and percolation fits in the middle panel, however, show that both germanium platforms have similar percolation densities ($1.4(1) \times 10^{10}$ cm^{-2} and $1.22(3) \times 10^{10}$ cm^{-2}, respectively). The percolation densities for holes in germanium-based platforms are significantly lower than the percolation densitis observed in ε-Si/SiGe ($6.9(1) \times 10^{10}$ cm^{-2}) and Si-MOS (1.86×10^{11} cm^{-2}).

In the right panel we evaluate the density-dependent transport scattering time $\tau_{tr} = m_0 m^* \mu / e$ to assess how differences in the effective mass m^* between platforms influence the electrical performance. For the germanium-based platforms, we used the respective density-dependent theoretical effective mass calculations $m^*(p)$ detailed in the main text, whereas for electrons in silicon we used a constant value of 0.19. At the benchmark density of 8×10^{10} cm^{-2} holes in Ge/ε-SiGe achieve a scattering time of ∼27 ps, which is about 5× shorter than the 140 ps achieved in ε-Ge/SiGe but much longer (∼ 18×) than the 1.5 ps in ε-Si/SiGe and

Si-MOS at peak. We speculate that the remaining factor of $\sim 5\times$ difference in scattering time between Ge/ε-SiGe and ε-Ge/SiGe is due to background impurity scattering from unwanted interfacial oxygen accumulation, a well-documented effect in Ge/SiGe heterostrucutres [4]. Notably, our measurements of interfacial oxygen concentration (2×10^{18} at/cm^3) is similar to the baseline levels reported in [4]. Since mitigating this contamination in [4] yielded a $4\times$ increase in mobility, we envision that successfully removing this oxygen in future epitaxial iterations will enhance our platform's scattering time to make it comparable to that of state of the art ε-Ge/SiGe reference.

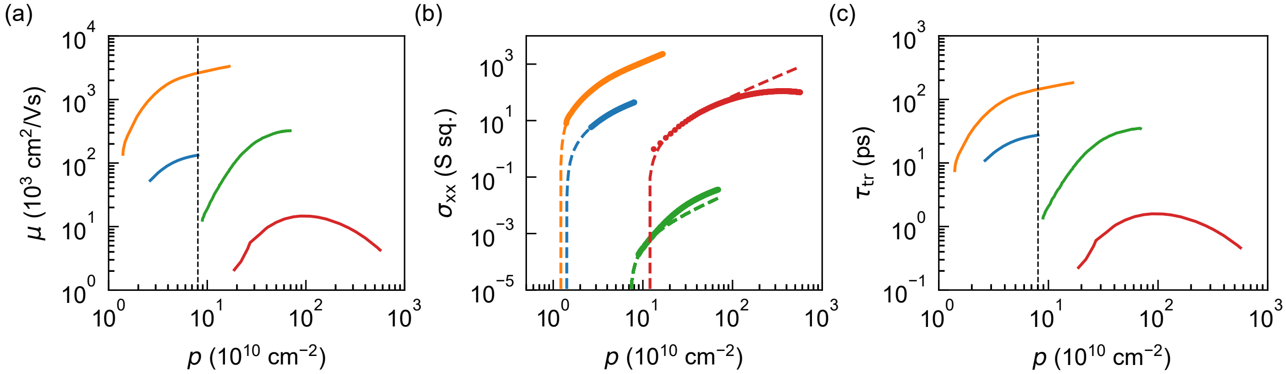

Supplementary Fig. 8. (a) Density-dependent mobility μ, (b) longitudinal conductivity σ_{xx}, and (c) transport scattering time τ_{tr} measured as a function of carrier density p. The curves compare ε-Ge/SiGe quantum wells [1] (orange), the Ge/ε-SiGe heterojunction presented in this work (blue), ε-Si/SiGe quantum wells [2] (green), and n-type Si-MOS [3] (red). In the middle panel, dashed lines represent fits to 2D percolation theory used to extract the critical percolation density p_{p} for each platform. In the left and right panels, the vertical dashed lines at 8×10^{10} cm^{-2} mark the carrier density at which the comparative scattering times are explicitly evaluated in the text.

1D quantization

Supplementary Fig. 9 displays the longitudinal conductance G_{xx} as a function of the applied split-gate voltage V_{sg}. Panel (a) shows the transport characteristics of our unstrained Ge/ε-SiGe heterojunction, exhibiting quantized steps for the lowest 1D subbands. For comparison, panel (b) displays the corresponding trace for the ε-Ge/SiGe quantum well, which resolves quantization steps up to higher subband indices. As the split gates are progressively energized to deplete the underlying 2D hole gas and electrostatically narrow the conduction channel, we observe distinct quantization plateaus at integer multiples of the quantum conductance $2e^2/h$, as direct evidence of ballistic 1D transport.

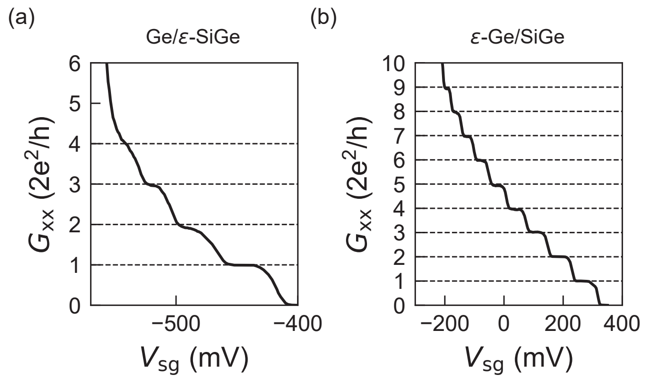

Supplementary Fig. 9. Longitudinal conductance G_{xx} measured in units of the quantum conductance $2e^2/h$ as a function of the applied constriction gate voltage V_{sg}. (a) Conductance trace for the Ge/ε-SiGe heterojunction, exhibiting distinct plateaus indicative of ballistic 1D transport. (b) Corresponding conductance trace for the ε-Ge/SiGe quantum well, showing well-resolved quantization steps up to higher 1D subband indices. In both panels, the horizontal dashed lines denote integer multiples of $2e^2/h$.

1D in-plane g-factor

The effective in-plane g-factor $g^*_{\parallel}$ was extracted from the evolution of spin-resolved 1D subbands under an applied in-plane magnetic field B. For each subband, the Zeeman splitting ΔE_Z was obtained from the separation in side-gate voltage V_{sg} between the corresponding transconductance peaks as a function of magnetic field B, converted into energy using the subband lever arm extracted from the source-drain bias spectroscopy. The effective g-factor was then obtained from the slope of the linear region of $\Delta E_Z(B)$ according to $g^*_{\parallel} = \Delta E_Z/\mu_{\mathrm{B}} B$, where μ_{B} is the Bohr magneton. The uncertainty was evaluated from the fitting error of the transconductance peak positions.

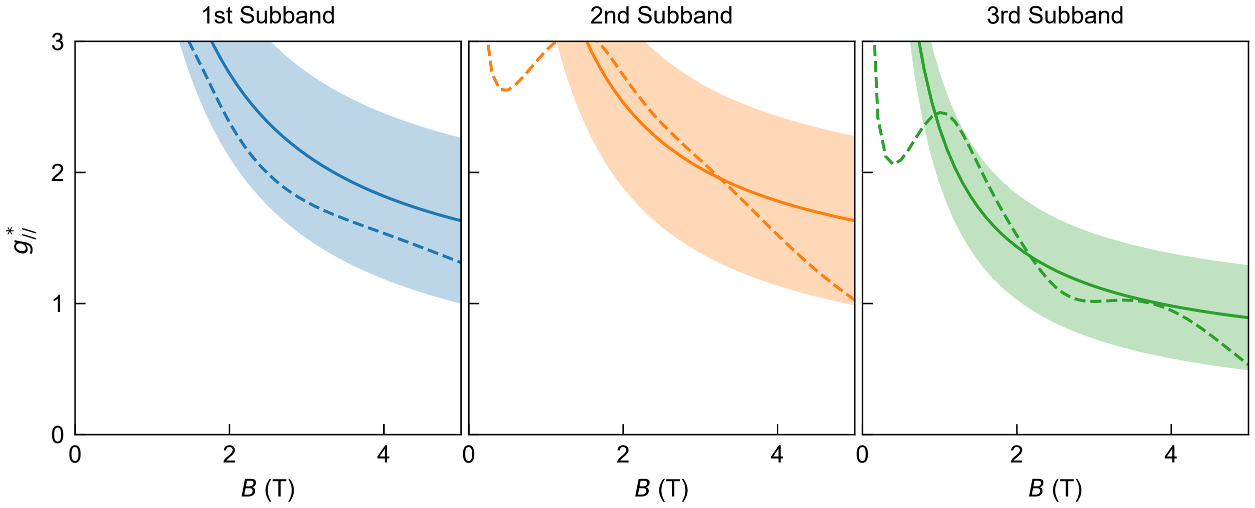

Supplementary Fig. 10. Experimental in-plane g-factor $g^*_{\parallel}$ (solid lines) and error (shaded regions) as a function of in-plane magnetic field B compared with theoretical simulations (dashed lines) for the first three 1D subbands. The measured data show a non-linear dependence on B, consistent with the field-dependent mixing of heavy-hole and light-hole states captured by the theoretical model.

THEORETICAL MODEL

Two-dimensional hole gas

We consider a two-dimensional hole gas (2DHG) in a Ge/SiGe heterostructure under the influence of a magnetic B and an electric field F_z, both applied to the z direction perpendicular to the 2-dimensional plane. We compare ε-Ge quantum wells and Ge/ε-SiGe strained-barrier heterojunctions with an unstrained Ge channel.

We use 6-band $k \cdot p$ theory to compute the energy levels and wavefunctions of the system. Because of quantum confinement in the 2DHG, we find different subbands (which we label by j) having a pseudospin degree of freedom (labelled by $\sigma = \pm 1$). To each of these levels is associated a 3-component spinor with envelope function components $f_j^{\nu}(z) \equiv \langle z | f_j^{\nu} \rangle$, where $\nu = \{\ell, s, h\}$ refer to LH, split-off hole and HH bands, respectively. For quantum wells grown on [001]-oriented substrates, and at $k_x = k_y = 0$ and $B = 0$, the eigenstates of the system are solutions of the following Hamiltonian [5]:

$$H_0^{\rm 2D} = \begin{bmatrix} H_{\sigma=+} & 0 \\ 0 & H_{\sigma=-} \end{bmatrix}, \tag{2a}$$

$$H_\sigma = H_\sigma^k + H_\sigma^\varepsilon + V, \tag{2b}$$

where

$$H_\sigma^k = \alpha_0 \begin{bmatrix} -k_z\gamma_+k_z & 2\sqrt{2}\sigma k_z\gamma_2 k_z & 0 \\ & -k_z\gamma_1 k_z & 0 \\ \dagger & & -k_z\gamma_- k_z \end{bmatrix}, \tag{3a}$$

$$H_\sigma^\varepsilon = a_v \mathrm{Tr}\,\varepsilon + b\cdot\delta\varepsilon \begin{bmatrix} -1 & \sqrt{2}\sigma & 0 \\ & 0 & 0 \\ \dagger & & 1 \end{bmatrix}, \tag{3b}$$

$$V = \mathcal{E}_{\Gamma_5^+} + \frac{\Delta_0}{3} + eF_z z - \Delta_0 \begin{bmatrix} 0 & 0 & 0 \\ & 1 & 0 \\ \dagger & & 0 \end{bmatrix}. \tag{3c}$$

Here, $\alpha_0 = \hbar^2/2m_0$, $\gamma_\pm = \gamma_1 \pm 2\gamma_2$ are Luttinger parameters, a_v and b are strain deformation potentials, $\delta\varepsilon = \varepsilon_{xx} - \varepsilon_{zz} \approx 1.67\varepsilon_{xx}$, where ε_{ij} is the strain tensor, Δ_0 is the bulk split-off gap, and $\mathcal{E}_{\Gamma_5^+}$ is the valence band edge energy without spin-orbit coupling. We consider $F_z = 0.1\,\mathrm{V}/\mu\mathrm{m}$ ($F_z = 0.05\,\mathrm{V}/\mu\mathrm{m}$) and $\varepsilon_{xx} = -0.857\%$ ($\varepsilon_{xx} = -0.018\%$) for the quantum well (heterojunction). The strain in the SiGe barriers is calculated assuming pseudomorphic growth. The energy band offsets between Ge and SiGe and the deformation potential constant b are obtained by linearly interpolating the values reported in reference [6]. The γ_i Luttinger parameters and g-factors are taken from reference [7]. The three γ_i and the κ parameter are interpolated in the full composition range according to the method outlined in reference [8]. We stress that each material parameter (γ_1, a_v, $\mathcal{E}_{\Gamma_5^+}$, ...) is a function of the position z in the heterostructure. Because of this spatial dependence, these parameters do not commute with k_z and we treat them as diagonal operators in Eq. (2a).

Focusing on the low-energy dynamics, the hole eigenvectors of the Hamiltonian H_0 in Eq. (2a) are either of pseudospin $\sigma = \pm 3/2$ (H levels) or pseudospin $\sigma = \pm 1/2$ (η levels) and they are explicitly given by

$$|\mathrm{H}\sigma; j\rangle = \left|\frac{3}{2}, \frac{3\sigma}{2}\right\rangle \left|f_j^h\right\rangle, \tag{4a}$$

$$|\eta\sigma; j\rangle = \left|\frac{3}{2}, \frac{\sigma}{2}\right\rangle \left|f_j^\ell\right\rangle + \sigma \left|\frac{1}{2}, \frac{\sigma}{2}\right\rangle \left|f_j^s\right\rangle, \tag{4b}$$

with corresponding σ-independent energies E_j^{H} and E_j^{η}, respectively. These eigenvectors comprise the bulk Bloch states $|J, M_J\rangle$ ($J = 3/2, 1/2$ and $M_J = 3\sigma/2, \sigma/2$) and spatially dependent smooth envelope functions $\left|f_j^h\right\rangle$, $\left|f_j^l\right\rangle$, $\left|f_j^s\right\rangle$. To compute these functions, we start from the spin up ($\sigma = +1$) block H_+ in (2a), which we diagonalize by finite differences methods using a z-mesh spacing of 0.01 nm and sharp interfaces between different material systems. The eigenstates of H_- are the time-reversal conjugates of the eigenstates of H_+.

Away from the Γ point (with $k_x = k_y = 0$), and including a finite out-of-plane magnetic field $\mathbf{B} = B\mathbf{e}_z$, the eigenstates of (2a) provide an orthonormal basis onto which the full $k\cdot p$ Hamiltonian can be projected. This results in (with bold characters indicating matrices expressed in the eigenbasis of H_0 and $\mathbf{K} = \mathbf{k} + e\mathbf{A}/\hbar$ is the dynamical momentum)

$$\mathbf{H}^{2\mathrm{D}} = \mathbf{E}_0^{2\mathrm{D}} + \alpha_0 \mathbf{M}_\gamma K_\parallel^2 + \frac{\alpha_0}{2l_B^2}\mathbf{M}_g + \alpha_0\left(i\mathbf{M}_1 K_- + \mathbf{M}_2 K_-^2 + \mathrm{h.c.}\right), \tag{5}$$

where $K_\pm = K_x \pm iK_y$, $K_\parallel^2 = K_x^2 + K_y^2 = \{K_-, K_+\}/2$, and

$$\mathbf{E}_0^{2\mathrm{D}} = \begin{bmatrix} \mathbf{E}^{\mathrm{H}} & 0 & 0 & 0 \\ & \mathbf{E}^{\eta} & 0 & 0 \\ & & \mathbf{E}^{\eta} & 0 \\ & & & \mathbf{E}^{\mathrm{H}} \end{bmatrix}, \tag{6}$$

with $\mathbf{E}^\tau = \mathrm{diag}\{E_1^\tau, E_2^\tau, \dots\}$ ($\tau = \{\eta, \mathrm{H}\}$) are the energies from (2a). The $\mathbf{M}$-matrices are

$$\mathbf{M}_\gamma = \begin{bmatrix} \boldsymbol{\Gamma}_\parallel^{\mathrm{H}} & 0 & 0 & 0 \\ & \boldsymbol{\Gamma}_\parallel^{\eta} & 0 & 0 \\ & & \boldsymbol{\Gamma}_\parallel^{\eta} & 0 \\ & & & \boldsymbol{\Gamma}_\parallel^{\mathrm{H}} \end{bmatrix}, \qquad \mathbf{M}_g = \begin{bmatrix} \mathbf{G}_\perp^{\mathrm{H}} & 0 & 0 & 0 \\ & \mathbf{G}_\perp^{\eta} & 0 & 0 \\ & & -\mathbf{G}_\perp^{\eta} & 0 \\ & & & -\mathbf{G}_\perp^{\mathrm{H}} \end{bmatrix}, \tag{7a}$$

$$\mathbf{M}_1 = \begin{bmatrix} 0 & \mathbf{T}^{\mathrm{x}} & 0 & 0 \\ 0 & 0 & \mathbf{T}^{\eta} & 0 \\ 0 & 0 & 0 & \mathbf{T}^{\mathrm{x}\dagger} \\ \mathbf{T}^{\mathrm{H}} & 0 & 0 & 0 \end{bmatrix}, \qquad \mathbf{M}_2 = \begin{bmatrix} 0 & 0 & \boldsymbol{\mu} & 0 \\ 0 & 0 & 0 & \boldsymbol{\mu}^\dagger \\ \boldsymbol{\delta}^\dagger & 0 & 0 & 0 \\ 0 & \boldsymbol{\delta} & 0 & 0 \end{bmatrix}. \tag{7b}$$

These matrix elements are explicitly expanded in terms of the eigenstates of (2a) as:

$$\boldsymbol{\Gamma}_\parallel^{\mathrm{H}} = -\langle f^h|\gamma_1 + \gamma_2|f^h\rangle\,, \tag{8a}$$

$$\mathbf{G}_\perp^{\mathrm{H}} = -\langle f^h|6\kappa + \frac{27q}{2}|f^h\rangle\,, \tag{8b}$$

$$\boldsymbol{\Gamma}_\parallel^{\eta} = -\langle f^z|\gamma_-|f^z\rangle - \langle f^\circ|\gamma_1 + \gamma_2|f^\circ\rangle\,, \tag{8c}$$

$$\mathbf{G}_\perp^{\eta} = -6\langle f^\circ|\kappa|f^\circ\rangle - \frac{1}{2}\langle f^\ell|q|f^\ell\rangle + 2\left(\langle f^z|f^z\rangle - 2\langle f^\circ|f^\circ\rangle\right), \tag{8d}$$

$$\mathbf{T}^{\mathrm{x}} = -\frac{3i}{\sqrt{2}}\langle f^h|\left(u_+\,|f^z\rangle + \frac{7\sqrt{6}}{12}[q, k_z]\,|f^\ell\rangle\right), \tag{8e}$$

$$\mathbf{T}^{\mathrm{H}} = -\frac{3i}{2}\langle f^h|[q, k_z]|f^h\rangle\,, \tag{8f}$$

$$\mathbf{T}^{\eta} = -\frac{3i}{\sqrt{2}}\left(\langle f^\circ|u_+|f^z\rangle - \langle f^z|u_-|f^\circ\rangle\right) - 5i\langle f^\ell|[q, k_z]|f^\ell\rangle\,, \tag{8g}$$

$$\boldsymbol{\mu} = \frac{3}{2}\langle f^h|\gamma_2 + \gamma_3|f^\circ\rangle\,, \tag{8h}$$

$$\boldsymbol{\delta} = \frac{3}{2}\langle f^h|\gamma_2 - \gamma_3|f^\circ\rangle\,, \tag{8i}$$

where $u_\pm = \{\gamma_3, k_z\} \pm [\kappa, k_z]$, $\{A, B\} \equiv AB + BA$ is the anti-commutator and

$$|f_j^z\rangle \equiv \frac{1}{\sqrt{3}}\left(\sqrt{2}\,|f_j^\ell\rangle - |f_j^s\rangle\right), \tag{9a}$$

$$|f_j^\circ\rangle \equiv \frac{1}{\sqrt{3}}\left(|f_j^\ell\rangle + \sqrt{2}\,|f_j^s\rangle\right). \tag{9b}$$

We omit for simplicity the explicit subband indices.

Landau levels

At large magnetic field values, we simulate the Landau level eigenspectrum as a function of the magnetic field applied out-of-plane.

In this case, the momenta in x and y direction do not commute and $[K_x, K_y] = -i\text{sign}(B)/l_B^2$, with $l_B = \sqrt{\hbar/e|B|} \sim 26$ nm at $B = 1$ T. Here, we consider positive values of B, and we define the dimensionless Landau level operators $a = iK_- l_B/\sqrt{2}$ and $a^\dagger = -iK_+ l_B/\sqrt{2}$, satisfying $[a, a^\dagger] = 1$. Note that the system has an additional degrees of freedom, the relative center of mass coordinate, which does not enter the Hamiltonian and thus produces the well-known degeneracy $\mathcal{N} = L_x L_y/2\pi l_B^2 = \phi/\phi_0$, coming by assuming the periodic boundary conditions in x and y directions. Here, ϕ is the magnetic flux in the sample of area $L_x L_y$ and ϕ_0 is the flux quantum.

We note that $K_\parallel^2 = (2a^\dagger a + 1)/l_B^2$, and that $\hbar^2/m_0 l_B^2 = 2\mu_B B$, such that the Hamiltonian in Eq. (5) reduces to

$$\tilde{\mathbf{H}} = \begin{bmatrix} \mathbf{E}_+^{\mathrm{H}}(B) & & & \\ & \mathbf{E}_+^{\eta}(B) & & \\ & & \mathbf{E}_-^{\eta}(B) & \\ & & & \mathbf{E}_-^{\mathrm{H}}(B) \end{bmatrix} + \frac{\alpha_0}{l_B^2} \begin{bmatrix} 2\mathbf{\Gamma}_\parallel^{\mathrm{H}} a^\dagger a & \sqrt{2} l_B \mathbf{T}^{\mathrm{x}} a & -2\boldsymbol{\mu} a^2 & \mathbf{0} \\ & 2\mathbf{\Gamma}_\parallel^{\eta} a^\dagger a & \sqrt{2} l_B \mathbf{T}^{\eta} a & -2\boldsymbol{\mu}^\dagger a^2 \\ \dagger & & 2\mathbf{\Gamma}_\parallel^{\eta} a^\dagger a & \sqrt{2} l_B \mathbf{T}^{\mathrm{x}\dagger} a \\ & & & 2\mathbf{\Gamma}_\parallel^{\mathrm{H}} a^\dagger a \end{bmatrix}, \tag{10}$$

where $\mathbf{E}_\pm^\tau(B) = \mathbf{E}^\tau + \frac{\alpha_0}{l_B^2}\left(\mathbf{\Gamma}_\parallel^\tau \pm \mathbf{G}_\perp^\tau/2\right)$ and we use the axial approximation $\gamma_2 = \gamma_3$. The distribution of the various powers of a and $a^\dagger$ in the Hamiltonian (10) suggests that all its eigenvectors $|\varphi\rangle$ can be written as

$$|\varphi\rangle = \begin{bmatrix} \mathbf{c}_{\mathrm{H}+} |m-1\rangle \\ \mathbf{c}_{\eta+} |m\rangle \\ \mathbf{c}_{\eta-} |m+1\rangle \\ \mathbf{c}_{\mathrm{H}-} |m+2\rangle \end{bmatrix}, \tag{11}$$

where the $\mathbf{c}_{\tau\pm}$ are scalars (one scalar for each H+ subband, and so on) and the $|m\rangle$ are the Fock states of the Landau levels operators, i.e. $a^\dagger a |m\rangle = |m\rangle m$. By projecting the Hamiltonian (10) onto the eigenvectors (11), we find a block diagonal Hamiltonian that involves only the integer m:

$$\tilde{\mathbf{H}}^{m\geq 1} = \begin{bmatrix} \mathbf{E}_+^{\mathrm{H}}(B) & & & \\ & \mathbf{E}_+^{\eta}(B) & & \\ & & \mathbf{E}_-^{\eta}(B) & \\ & & & \mathbf{E}_-^{\mathrm{H}}(B) \end{bmatrix} + \frac{\alpha_0}{l_B^2} \begin{bmatrix} 2\mathbf{\Gamma}_\parallel^{\mathrm{H}}(m-1) & \sqrt{2} l_B \mathbf{T}^{\mathrm{x}} \sqrt{m} & -2\boldsymbol{\mu}\sqrt{m(m+1)} & \mathbf{0} \\ & 2\mathbf{\Gamma}_\parallel^{\eta} m & \sqrt{2} l_B \mathbf{T}^{\eta}\sqrt{m+1} & -2\boldsymbol{\mu}^\dagger\sqrt{(m+1)(m+2)} \\ & & 2\mathbf{\Gamma}_\parallel^{\eta}(m+1) & \sqrt{2} l_B \mathbf{T}^{\mathrm{x}\dagger}\sqrt{m+2} \\ & & & 2\mathbf{\Gamma}_\parallel^{\mathrm{H}}(m+2) \end{bmatrix}. \tag{12}$$

This Hamiltonian can be diagonalized to extract the set of scalars $\mathbf{c}_{\tau\sigma}$ for any $m \geq -2$. For $m = -2$, only the H$-$ part of $|\varphi\rangle$ is well defined (since $|m+2\rangle = |0\rangle$), and implies that only the scalars $\mathbf{c}_{\mathrm{H}-}$ are non-zero. For $m = -1$, the H$-$ as well as the η$-$ subspaces are well-defined, and only $\mathbf{c}_{\mathrm{H}-}$ and $\mathbf{c}_{\eta-}$ are non-zero. Similarly, the three subspaces H$-$, η$-$ and η$+$ are well defined for $m = 0$, and therefore $\mathbf{c}_{\mathrm{H}+}$ must be zero. For any $m \geq 1$, the Hamiltonian (12) is diagonalized directly without any conditions on $\mathbf{c}_{\tau\sigma}$. We remark that this procedure is independent of the gauge.

We truncate the system to include only the levels from $m = -2$ to $m = 11$ and vary B. This gives a total of $2 \times 11 + 3 = 25$ magnetic field-dependent spin-polarized Landau levels for each HH subband in the z-direction, which we now label by $n \geq 1$, each one degenerate by $\mathcal{N}$ because of the residual center of mass degree of freedom.

The dependence on B of the numerically computed Landau level energies ϵ_n (labelled in increasing order of energy) enables us to extract the B-field-dependent effective spin-resolved mass and g factor of the hole gas as

$$\frac{1}{m^*_\downarrow} = \frac{\epsilon_{n=3} - \epsilon_{n=1}}{2\mu_B B}\ , \qquad \frac{1}{m^*_\uparrow} = \frac{\epsilon_{n=4} - \epsilon_{n=2}}{2\mu_B B}\ , \text{ and } \ g^*_\perp = \frac{\epsilon_{n=2} - \epsilon_{n=1}}{\mu_B B}\ . \tag{13}$$

The density p is related to the magnetic field via the filling factor $\nu = 2\pi l_B^2 p$, which then results in $B = b_0 p/\nu$, with $b_0 = h/e = 4.13\ \mathrm{T}/(10^{11}\ \mathrm{cm}^{-2})$. This definition of g factor and effective mass is consistent with thermal activation energy measurements, probing the activation energy of the $\nu = 1$ ($\nu = 2$) filling factor for $m^*_\downarrow$ and $g^*_\perp$ ($m^*_\uparrow$). The plots in the main text display the extracted spin down mass $m^*_\downarrow$ and g-factor obtained from (13). We exclude from the plots the additional levels with $n > 4$.

1D channels

We model strained and unstrained Ge QPCs by introducing an in-plane confinement profile $V_\parallel(x)$ to the $k \cdot p$ Hamiltonian, where x is the direction of quantization and y is parallel to the direction of motion. We describe magnetic fields directed towards the y-axis with the Landau gauge

$$\mathbf{A}(\mathbf{r}) = -Bx\mathbf{e}_z, \tag{14}$$

such that $\nabla \times \mathbf{A} = B\mathbf{e}_y$. In the unstrained Ge QPC the measured subband energy splitting is relatively constant, indicating that an harmonic confinement suffices to describe the data and avoids over-fitting. Therefore, in this case, we use a simple parabolic confinement $V^{\mathrm{u}}_\parallel(x) = -\alpha_0 x^2/\ell^4$ with $\ell = 28\,\mathrm{nm}$ and where the minus sign comes from our chosen hole-energy convention. In the strained Ge QPC, where the measured subband gap is largely anharmonic, we model the confinement with

$$V^{\mathrm{s}}_\parallel(x) = -V_0 \left[1 - \frac{1}{\sqrt{1 + 2\alpha_0 x^2/(V_0 \ell^4)}}\right], \tag{15}$$

where $\ell = 9.9\,\mathrm{nm}$ and $V_0 = 13.5\,\mathrm{meV}$. This confinement potential nicely reproduces the $\sim 1/n$ trend of the higher energy gaps. Note that $V^{\mathrm{s}}_\parallel(|x| \to \infty) = -V_0$ and $V^{\mathrm{s}}_\parallel(x) \simeq -\alpha_0 x^2/\ell^4$ when $x \ll \ell^2\sqrt{V_0/(2\alpha_0)}$. We remark that $\alpha_0 = \hbar^2/(2m_0)$, i.e. the effective parabolic length ℓ is related to that of a free electron of mass m_0. The strained Ge QPC Hamiltonian is solved in position space by means of finite differences with $k_x = -i\partial_x$, a mesh step $\delta x = 1\,\mathrm{nm}$ and

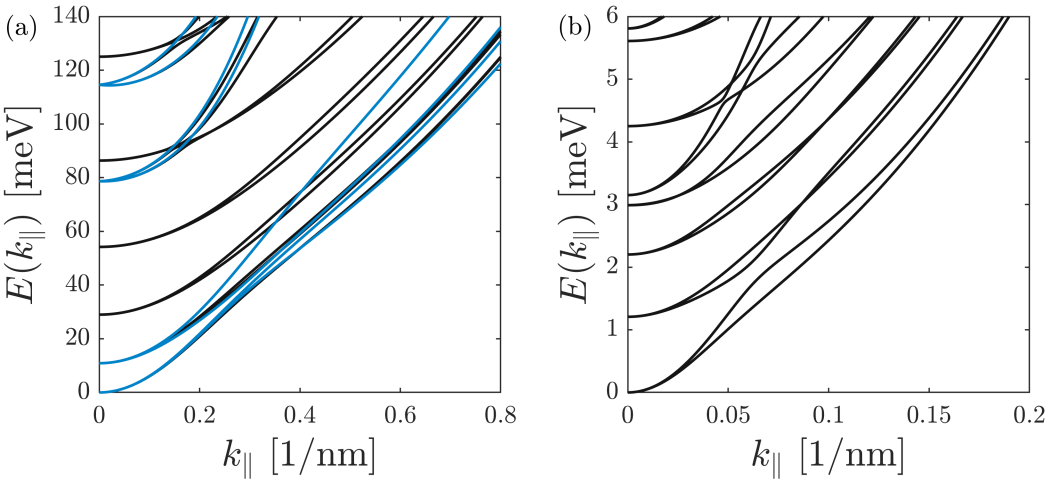

Supplementary Fig. 11. Energy dispersion $E(k_\parallel)$ of the (a) strained Ge QW computed from the full 200-subband matrix (black) and from the effective 4-band matrix (blue) and (b) of the unstrained Ge system computed from the 200-subband matrix.

in the domain $x \in [-750, +750]$ nm. The unstrained Ge QPC Hamiltonian is projected onto the first 30 eigenstates of the harmonic oscillator, obtained by writing $x = \frac{\ell}{\sqrt{2}}\left(a + a^\dagger\right)$ and $k_x = \frac{1}{\sqrt{2}i\ell}\left(a - a^\dagger\right)$, where a is the usual ladder operator.

We first diagonalize the 1D channel Hamiltonian $\mathbf{H}_0^{1\mathrm{D}}$ at $B = 0$ and $k_y = 0$. This later provides an orthonormal basis on which the full Hamiltonian at finite B and k_y is projected. This Hamiltonian is

$$\mathbf{H}_0^{1\mathrm{D}} = \mathbf{E}_0^{2\mathrm{D}} + \alpha_0 \left[\left(\mathbf{M}_\gamma + \mathbf{M}_2 + \mathbf{M}_2^\dagger \right) k_x^2 + i \left(\mathbf{M}_1 - \mathbf{M}_1^\dagger \right) k_x \right] + V_\parallel(x), \tag{16}$$

where $\mathbf{E}_0^{2\mathrm{D}}$ are the energies of the planar system without in-plane confinement. For unstrained Ge, the $\mathbf{M}$-matrices correspond to those defined in Eq. (7), truncated to include the first 200 2D subbands. This results in a Hamiltonian of dimension $2 \times 30 \times 200 = 12000$. In strained Ge, given the large basis along the x-dimension, we collapse the 2D basis down onto the first 2 HH levels + the first 2 LH levels, with the remaining $200 - 4 = 196$ subbands are included by a 2nd order perturbation. The ground state dispersion from the 4-subband Hamiltonian is in good agreement with that from the complete 200-subband Hamiltonian over a range of wavenumber that is well beyond the requirements of the following calculations ($\sim 1/(10\,\mathrm{nm}) = 0.1\,\mathrm{nm}^{-1}$, see Supplementary Fig. 11). The resulting 1D Hamiltonian is of dimension $2 \times 1501 \times 4 = 12008$ and has the same structure as (16), but with renormalized $\mathbf{M}$-matrices:

$$\tilde{\mathbf{M}}_1 = \mathbf{M}_1, \tag{17a}$$

$$\tilde{M}_{\gamma,j}^i = M_{\gamma,j}^i + \alpha_0 \sum_{k\in\mathcal{B}} \left(\frac{M_{1,k}^i M_{1,j}^{\dagger k}}{E_i - E_k} + \frac{M_{1,k}^i M_{1,j}^{\dagger k}}{E_j - E_k} + \frac{M_{1,k}^{\dagger i} M_{1,j}^k}{E_i - E_k} + \frac{M_{1,k}^{\dagger i} M_{1,j}^k}{E_j - E_k} \right), \tag{17b}$$

$$\tilde{M}_{2,j}^i = M_{2,j}^i - \alpha_0 \sum_{k\in\mathcal{B}} \left(\frac{M_{1,k}^i M_{1,j}^k}{E_i - E_k} + \frac{M_{1,k}^i M_{1,j}^k}{E_j - E_k} \right), \tag{17c}$$

where the $\mathcal{B}$-set refers to the remote 196 subbands. The Hamiltonian $\mathbf{H}_0^{1\mathrm{D}}$ is diagonalized for the first 501 channel subbands for unstrained Ge and for the first 540 subbands for strained Ge (not counting spin in both cases). Then the full 1D channel Hamiltonian is projected onto the 1D channel basis states, and diagonalized at finite B and k_y. The result is

$$\mathbf{H}^{1\mathrm{D}} = \mathbf{E}_0^{1\mathrm{D}} + \alpha_0 \left(\mathbf{L}_1 k_y + \mathbf{L}_\gamma k_y^2 + \frac{1}{2l_B^2}\mathbf{L}_{g\parallel} + \frac{1}{2l_B^2}\mathbf{L}_{3\parallel} k_y + \frac{1}{4l_B^4}\mathbf{L}_{4\parallel} \right), \tag{18}$$

where the $\mathbf{L}$-matrices are

$$L^i_{1,j} = v^{\dagger i}{}_k \left[m^k_{1,l} - 2(\mathbf{m}_2 k_x)^k{}_l \right] v^l{}_j, \tag{19a}$$
$$L^i_{\gamma,j} = v^{\dagger i}{}_k \left[m^k_{0,l} \right] v^l{}_j, \tag{19b}$$
$$L^i_{g\parallel,j} = v^{\dagger i}{}_k \left[n^k_{g,l} - (\mathbf{N}'_\gamma x)^k{}_l + (\mathbf{n}'_1 \{x, k_x\})^k{}_l \right] v^l{}_j, \tag{19c}$$
$$L^i_{3\parallel,j} = v^{\dagger i}{}_k \left[2(\mathbf{n}_1 x)^k{}_l \right] v^l{}_j, \tag{19d}$$
$$L^i_{4\parallel,j} = v^{\dagger i}{}_k \left[(\mathbf{N}_\gamma x^2)^k{}_l \right] v^l{}_j, \tag{19e}$$
$$\tag{19f}$$

with $v^i{}_j$ being the i-th component of the j-th eigenvector of $\mathbf{H}_0^{1\mathrm{D}}$ (i.e. $v^{\dagger i}{}_k v^k{}_j = \delta^i_j$) and where

$$\mathbf{m}_0 = \mathbf{M}_\gamma - \mathbf{M}_2 - \mathbf{M}_2^\dagger, \tag{20a}$$
$$\mathbf{m}_1 = \mathbf{M}_1 + \mathbf{M}_1^\dagger, \tag{20b}$$
$$\mathbf{m}_2 = i\left(\mathbf{M}_2 - \mathbf{M}_2^\dagger\right), \tag{20c}$$
$$\mathbf{n}_g = -i\left(\mathbf{N}_g - \mathbf{N}_g^\dagger\right), \tag{20d}$$
$$\mathbf{n}_1 = \mathbf{N}_1 + \mathbf{N}_1^\dagger, \tag{20e}$$
$$\mathbf{n}'_1 = i\left(\mathbf{N}_1 - \mathbf{N}_1^\dagger\right). \tag{20f}$$

The $\mathbf{N}$-matrices describe the 2D system with in-plane magnetic fields:

$$\mathbf{N}_g = \begin{bmatrix} 0 & \mathbf{G}_\parallel^\times & 0 & 0 \\ 0 & 0 & \mathbf{G}_\parallel^\eta & 0 \\ 0 & 0 & 0 & \mathbf{G}_\parallel^{\times\dagger} \\ 0 & 0 & 0 & 0 \end{bmatrix}, \qquad \mathbf{N}_1 = \begin{bmatrix} 0 & \mathbf{R}^\times & 0 & 0 \\ 0 & 0 & \mathbf{R}^\eta & 0 \\ 0 & 0 & 0 & \mathbf{R}^{\times\dagger} \\ 0 & 0 & 0 & 0 \end{bmatrix}, \tag{21}$$

$$\mathbf{N}_\gamma = \begin{bmatrix} \boldsymbol{\Gamma}_\perp^{\mathrm{H}} & 0 & 0 & 0 \\ 0 & \boldsymbol{\Gamma}_\perp^\eta & 0 & 0 \\ 0 & 0 & \boldsymbol{\Gamma}_\perp^\eta & 0 \\ 0 & 0 & 0 & \boldsymbol{\Gamma}_\perp^{\mathrm{H}} \end{bmatrix}, \qquad \mathbf{N}'_\gamma = \begin{bmatrix} \boldsymbol{\Gamma}_\perp^{\prime\mathrm{H}} & 0 & 0 & 0 \\ 0 & \boldsymbol{\Gamma}_\perp^{\prime\eta} & 0 & 0 \\ 0 & 0 & \boldsymbol{\Gamma}_\perp^{\prime\eta} & 0 \\ 0 & 0 & 0 & \boldsymbol{\Gamma}_\perp^{\prime\mathrm{H}} \end{bmatrix}, \tag{22}$$

where

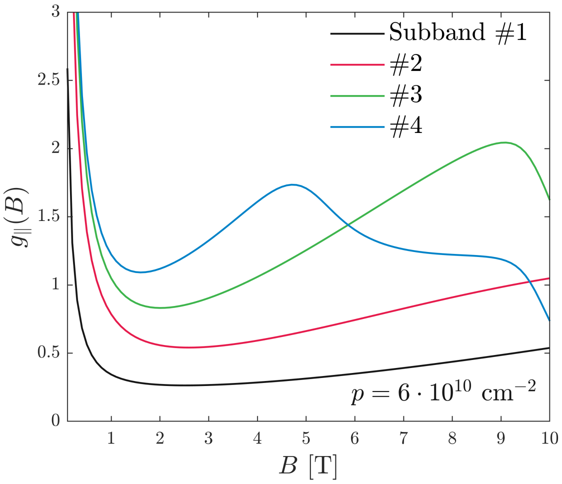

Supplementary Fig. 12. Calculated in-plane g-factor of the first 4 1D subbands as a function of B for the strained Ge QPC.

$$\mathbf{G}_\parallel^\eta = -\sqrt{2}\left(\langle f^\circ|\, 3\kappa + 1\, |f^z\rangle + \langle f^z|\, 3\kappa + 1\, |f^\circ\rangle\right) + 2\,\langle f^z|f^z\rangle\,, \tag{23a}$$

$$\mathbf{G}_\parallel^\times = -\sqrt{2}\,\langle f^h| \left(3\kappa\, |f^z\rangle - \sqrt{3}\, |f^s\rangle\right), \tag{23b}$$

$$\mathbf{R}^\eta = 3\sqrt{2}i\left(\langle f^\circ|\, \gamma_3\, |f^z\rangle - \langle f^z|\, \gamma_3\, |f^\circ\rangle\right), \tag{23c}$$

$$\mathbf{R}^\times = 3\sqrt{2}i\,\langle f^h|\, \gamma_3\, |f^z\rangle\,, \tag{23d}$$

$$\mathbf{\Gamma}_\perp^\mathrm{H} = -\,\langle f^h|\, \gamma_1 - 2\gamma_2\, |f^h\rangle\,, \tag{23e}$$

$$\mathbf{\Gamma}_\perp^{\prime\mathrm{H}} = -\frac{1}{2}\,\langle f^h|\, \{\gamma_1 - 2\gamma_2, k_z\}\, |f^h\rangle\,, \tag{23f}$$

$$\mathbf{\Gamma}_\perp^\eta = -\,\langle f^\circ|\, \gamma_1 - 2\gamma_2\, |f^\circ\rangle - \langle f^z|\, \gamma_1 + 4\gamma_2\, |f^z\rangle\,, \tag{23g}$$

$$\mathbf{\Gamma}_\perp^{\prime\eta} = -\frac{1}{2}\,\langle f^\circ|\, \{\gamma_1 - 2\gamma_2, k_z\}\, |f^\circ\rangle - \frac{1}{2}\,\langle f^z|\, \{\gamma_1 + 4\gamma_2, k_z\}\, |f^z\rangle\,. \tag{23h}$$

The Zeeman splitting as a function of B is then computed for fixed $k_y = \sqrt{2\pi p}$, determined by the reported hole density for each experiment: $p = 5 \times 10^{10}\,\mathrm{cm}^{-2}$ in the unstrained Ge channel and $p = 6 \times 10^{10}\,\mathrm{cm}^{-2}$ in the strained Ge channel. The computed g-factor $g_\parallel = \Delta E/\mu_\mathrm{B} B$ for the unstrained Ge QPC is plotted against the g-factor extracted from experiment in Supplementary Fig. 6 (dashed lines). The computed g-factor as a function of B for the strained Ge QPC is plotted in Supplementary Fig. 12.

* g.scappucci@tudelft.nl

[1] L. E. A. Stehouwer, A. Tosato, D. Degli Esposti, D. Costa, M. Veldhorst, A. Sammak, and G. Scappucci, Applied Physics Letters **123**, 092101 (2023).

[2] D. Degli Esposti, L. E. A. Stehouwer, O. Gül, N. Samkharadze, C. Déprez, M. Meyer, I. N. Meijer, L. Tryputen, S. Karwal, M. Botifoll, J. Arbiol, S. V. Amitonov, L. M. K. Vander-

sypen, A. Sammak, M. Veldhorst, and G. Scappucci, npj Quantum Information **10** (2024), 10.1038/s41534-024-00826-9.

[3] T. N. Camenzind, A. Elsayed, F. A. Mohiyaddin, R. Li, S. Kubicek, J. Jussot, P. Van Dorpe, B. Govoreanu, I. Radu, and D. M. Zumbühl, Materials for Quantum Technology **1**, 041001 (2021).

[4] J. Lu, J. Li, H. Wang, W. Bian, Y. Zhang, T. Pei, and J. Luo, Chinese Physics B (2026), 10.1088/1674-1056/ae39d4.

[5] P. Del Vecchio and O. Moutanabbir, Phys. Rev. B **110**, 045409 (2024).

[6] C. G. Van de Walle and R. M. Martin, Phys. Rev. B **34**, 5621 (1986).

[7] R. Winkler, *Spin-orbit Coupling Effects in Two-Dimensional Electron and Hole Systems*, Vol. 191 (Springer, 2003).

[8] R. Winkler, M. Merkler, T. Darnhofer, and U. Rössler, Phys. Rev. B **53**, 10858 (1996).
